# Balancing selection on an MYB transcription factor maintains the twig trichome color variation in *Melastoma normale*

**DOI:** 10.1186/s12915-023-01611-4

**Published:** 2023-05-24

**Authors:** Guilian Huang, Wei Wu, Yongmei Chen, Xueke Zhi, Peishan Zou, Zulin Ning, Qiang Fan, Ying Liu, Shulin Deng, Kai Zeng, Renchao Zhou

**Affiliations:** 1 State Key Laboratory of Biocontrol and Guangdong Provincial Key Laboratory of Plant Resources, School of Life Sciences, Sun Yat-Sen University, Guangzhou, 510275 China; 2 College of Chemical Engineering, Sichuan University of Science & Engineering, Zigong, Sichuan 643000 China; 3 Guangdong Provincial Key Laboratory of Applied Botany, South China Botanical Garden, Chinese Academy of Sciences, Guangzhou, 510650 China; 4 Department of Animal and Plant Sciences, University of Sheffield, Sheffield, UK

**Keywords:** Genetic basis, Balancing selection, Color variation, Introgression, *Melastoma*

## Abstract

**Background:**

The factors that maintain phenotypic and genetic variation within a population have received long-term attention in evolutionary biology. Here the genetic basis and evolution of the geographically widespread variation in twig trichome color (from red to white) in a shrub *Melastoma normale* was investigated using Pool-seq and evolutionary analyses.

**Results:**

The results show that the twig trichome coloration is under selection in different light environments and that a 6-kb region containing an R2R3 MYB transcription factor gene is the major region of divergence between the extreme red and white morphs. This gene has two highly divergent groups of alleles, one of which likely originated from introgression from another species in this genus and has risen to high frequency (> 0.6) within each of the three populations under investigation. In contrast, polymorphisms in other regions of the genome show no sign of differentiation between the two morphs, suggesting that genomic patterns of diversity have been shaped by homogenizing gene flow. Population genetics analysis reveals signals of balancing selection acting on this gene, and it is suggested that spatially varying selection is the most likely mechanism of balancing selection in this case.

**Conclusions:**

This study demonstrate that polymorphisms on a single transcription factor gene largely confer the twig trichome color variation in *M. normale*, while also explaining how adaptive divergence can occur and be maintained in the face of gene flow.

**Supplementary Information:**

The online version contains supplementary material available at 10.1186/s12915-023-01611-4.

## Background

Both phenotypic and genetic variations are critical for organismal survival, evolution, and adaptation [1, 2]. The factors that maintain phenotypic and genetic variation within a population have received long-term attention in evolutionary biology [3]. While positive directional selection, negative selection, and genetic drift are all expected to eventually reduce genetic variation [4, 5], balancing selection, on the other hand, is often considered a possible mechanism for maintaining variation [4, 6]. Balancing selection can result from spatially or temporally varying selection, heterozygote advantage, or negative frequency dependent selection (reviewed in [6–8]). Examples of all three forms of balancing selection have been reported before [9–13].

Dissecting the genetic basis of variable traits may provide insight into fundamental questions about the origin, evolution, and maintenance of variation within populations [12, 14]. However, finding suitable candidate genes for studying balancing selection is difficult, because genetic basis of traits of interest is usually poorly characterized [6, 15]. Genomic scans are now increasingly used to detect balancing selection for inferring its role in the maintenance of genetic variation and this kind of analysis has been conducted in humans [16–21], bacteria [22, 23], *Drosophila* [24], human malaria parasite [25], and plants [26, 27]. In plants, well known examples of balancing selection are disease resistance genes [28–30], self-incompatibility genes [31, 32], and genes related to response to biotic and abiotic stresses [11, 13, 26].

*Melastoma*, a shrub genus consisting of 80–100 species in tropical Asia and Oceania [33, 34], has experienced rapid species radiation in the past one million years [35]. Trichomes on leaf, twig, and hypanthium are the most important morphological traits for species delimitation in *Melastoma* [34, 36]. Most species in this genus possess appressed scales on twigs, while a few species exhibit spreading hairs or bristles on twigs [34, 36]. Species of *Melastoma* show differential adaptation to heterogeneous environments with respect to light, temperature, water, and soil conditions [34], offering an excellent system to study the genetic basis of adaptation. The genome sequence of *Melastoma candidum*, a common species in southern China and northern Vietnam, has recently become available, facilitating the identification and characterization of genes and genomic regions underlying adaptation and speciation in this genus.

**Fig. 1:**
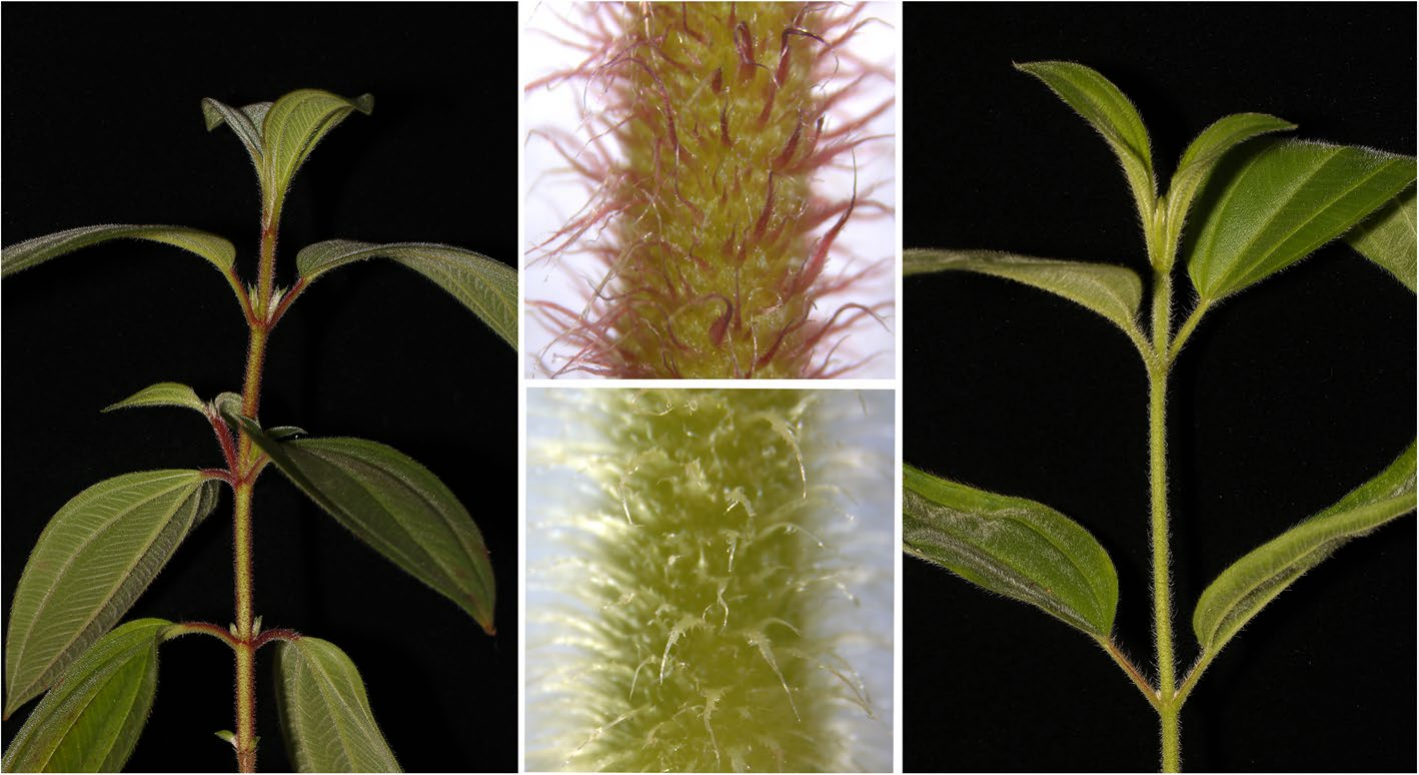
Trichomes on twigs of *Melastoma normale*. Shown on the left and right panels are the red and white morphs of *M. normale*, respectively. Only red and white morphs are shown here. Details of twig trichomes of the red (upper) and white (lower) morphs are shown on the middle panel

*Melastoma normale* is one of the most widespread species in this genus, ranging from the edges of the Himalaya (Nepal, Bhutan, and India) across northern Southeastern Asia (northern Thailand, Laos, and Vietnam) to southern China [33, 36]. It displays continuous twig trichome color variation from red to white within populations (Fig. 1). In the wild, individuals with red twig trichomes prefer open habitats with high sunlight intensity, those with white twig trichomes are mostly found in slightly shady habitats with relatively low sunlight intensity, and those with twig trichomes of intermediate colors are mostly seen in the intermediate habitats. Hereafter, individuals with extreme red and white twig trichomes were called as Red morph and White morph, respectively. The two morphs of *M. normale* are widespread within populations and can be observed throughout its geographic range. Trichomes in plants play numerous adaptive roles, including defense against biotic attack (e.g., insect herbivores and fungal pathogens) and mitigation of abiotic stress (e.g., water evaporation and UV radiation) [14, 37]. Given that anthocyanin and other flavonoids play important roles in plant photoprotection [38], red trichomes of *M. normale* might be more tolerant to high light conditions and have a selective advantage in open habitats, whereas white trichomes may have a selective advantage in slightly shady habitats as it will not invest additional energy to synthesize anthocyanins to ensure more investment on growth and reproduction. Thus, red and white trichomes on the twigs of *M. normale* may be a plausible adaptation to UV radiation associated with their exposed or slightly shady habitats, respectively. It is possible that twig trichome coloration in *M. normale* is a phenotypically plastic trait, as vegetative organs of many plants can accumulate anthocyanin at high light conditions (e.g., [39, 40]). However, the common garden experiment (see below) suggests that twig trichome color variation in *M. normale* is largely genetically controlled.

To date, both the genetic basis of trichome color variation in *M. normale*, and the evolutionary processes involved in its origin and maintenance have remained unknown. The wide accessibility of high-throughput sequencing technology makes it possible to identify genomic regions underlying traits of interest in natural populations using population genomic scan approaches (e.g., [12, 14]). These approaches require low levels of neutral differentiation between populations (or ecotypes) in order to distinguish signal from background differentiation [41]. The microhabitat divergence of the two morphs of *M. normale* in the face of gene flow is fairly suitable for genomic scan approaches because local differentiation with substantial gene flow should proceed primarily through mutations of major effect [42]. In this study, it is hypothesized that twig trichome coloration of *M. normale* is under selection in different light conditions and that the underlying gene(s) is(are) under balancing selection to maintain the twig trichome color variation. To this end, a common garden experiment was done to test the first hypothesis, a Pool-seq approach was used to dissect the genetic basis of twig trichome color variation, and some evolutionary analyses were performed to infer the origin of genetic variation underlying this phenotypic variation, and to test if balancing selection maintains this variation. The results show that the widespread twig trichome color variation in *M. normale* is under selection in different light environments and involves largely a R2R3 MYB transcription factor under balancing selection. The gene has two highly divergent groups of alleles, one of which likely originated from introgression from another species.

## Results

### Twig trichome coloration in *M. normale* is under selection in different light environments

**Fig. 2:**
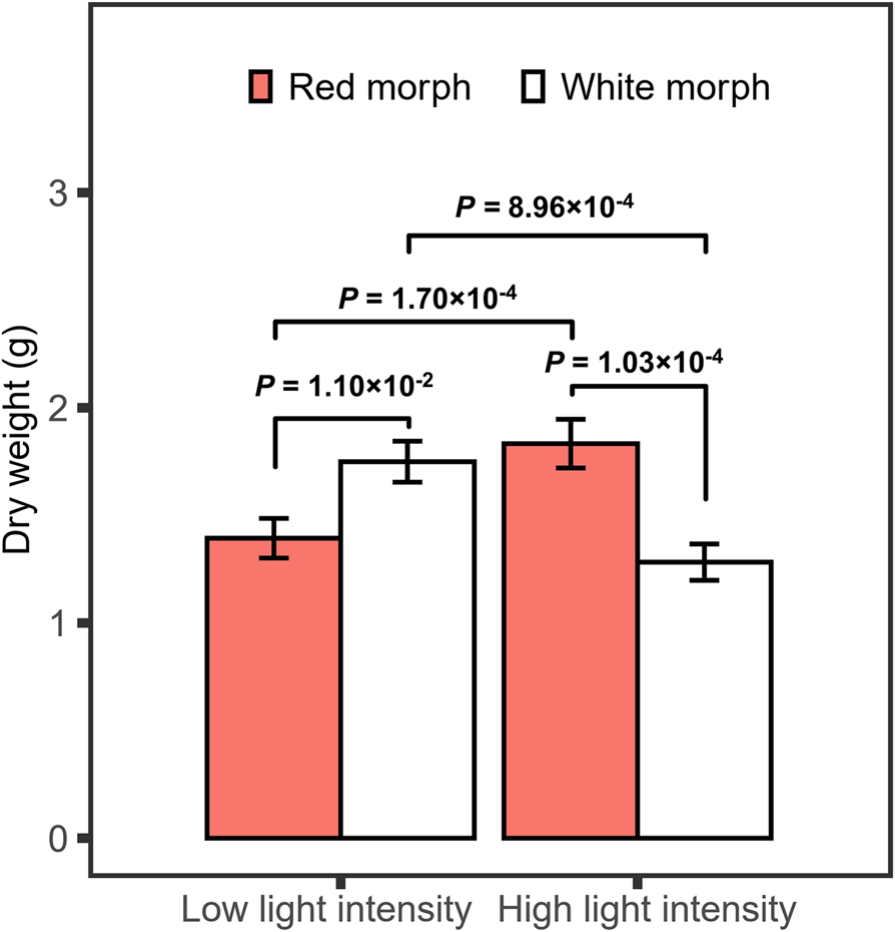
Pairwise comparison of plant dry weight for two morphs of *Melastoma normale*. Plant dry weight was measured for 30 one-year-old individuals each at high and low sunlight intensities of each morph in a common garden experiment. Statistical significance was determined with the LSD test using SPSS UNIANOVA

A common garden experiment was used to test if twig trichome coloration of *M. normale* is under selection in different light environments (see “Methods” for details). For each of the Red and White morphs, plant dry weights were measured for 30 one-year-old individuals each at high and low sunlight intensities. Because there was a statistically significant interaction between the effect of morph and sunlight intensity (two-way ANOVA tests, F_1, 116_ = 21.90, *p* = 8.70 × 10^–6^), pairwise comparison for the two morphs was made at high and low sunlight intensities, respectively. As shown in Fig. 2, plants of the Red morph had significantly higher dry weight at high sunlight intensity than at low sunlight intensity. In contrast, plants of the White morph showed significantly higher dry weight at low sunlight intensity than at high sunlight intensity. At low sunlight intensity, plants of the White morph had significantly higher dry weight than plants of the Red morph. By contrast, at high sunlight intensity, plants of the Red morph had significantly higher dry weight than plants of the White morph. The contrast in biomass accumulation between the Red and White morphs suggests that twig trichome coloration in *M. normale* is under selection in different light environments. Meanwhile, it was found that white twig trichomes would not change to red twig trichomes at high sunlight condition, and red twig trichomes would not change to white twig trichomes at low sunlight condition, suggesting that twig trichome color variation in *M. normale* is largely, if not completely, genetically controlled.

### An MYB transcription factor is the major gene associated with twig trichome color variation in *M. normale*

To identify the genetic basis of twig trichome color variation in *M. normale*, 24–30 individuals for each morph with the extreme trichome colors were sampled from each of the two locations of Guangzhou, Dishuiyan (Panyu District), and Maofengshan (Baiyun District), with a distance of about 70 km between them (Additional file 1: Table S1). The samples of each morph from each sampling location were pooled and sequenced on an Illumina Hiseq 2500 platform. After quality filtering, 226.6–246.7 million paired end reads (~ 126–137 × in depth) were obtained for the four pooled samples and more than 95% of these reads were mapped onto the reference genome of *M. candidum*, a closely related species of *M. normal*e (Additional file 1: Table S2).

Using a 5-kb sliding window analysis, the average nucleotide diversity (*π*) and nucleotide polymorphism (*θ*_w_) is highly similar among the four pooled samples (Additional file 1: Figs. S1 and S2), and the two morphs in the same sampling location had nearly identical average nucleotide diversity (0.0082 and 0.0083 in Dishuiyan; 0.0093 and 0.0094 in Maofengshan; Additional file 1: Table S3). The average Tajima’s D values were all below 0 in the four samples, and again, the two morphs in the same sampling location had nearly identical values (Additional file 1: Table S3, Fig. S3).

**Fig. 3:**
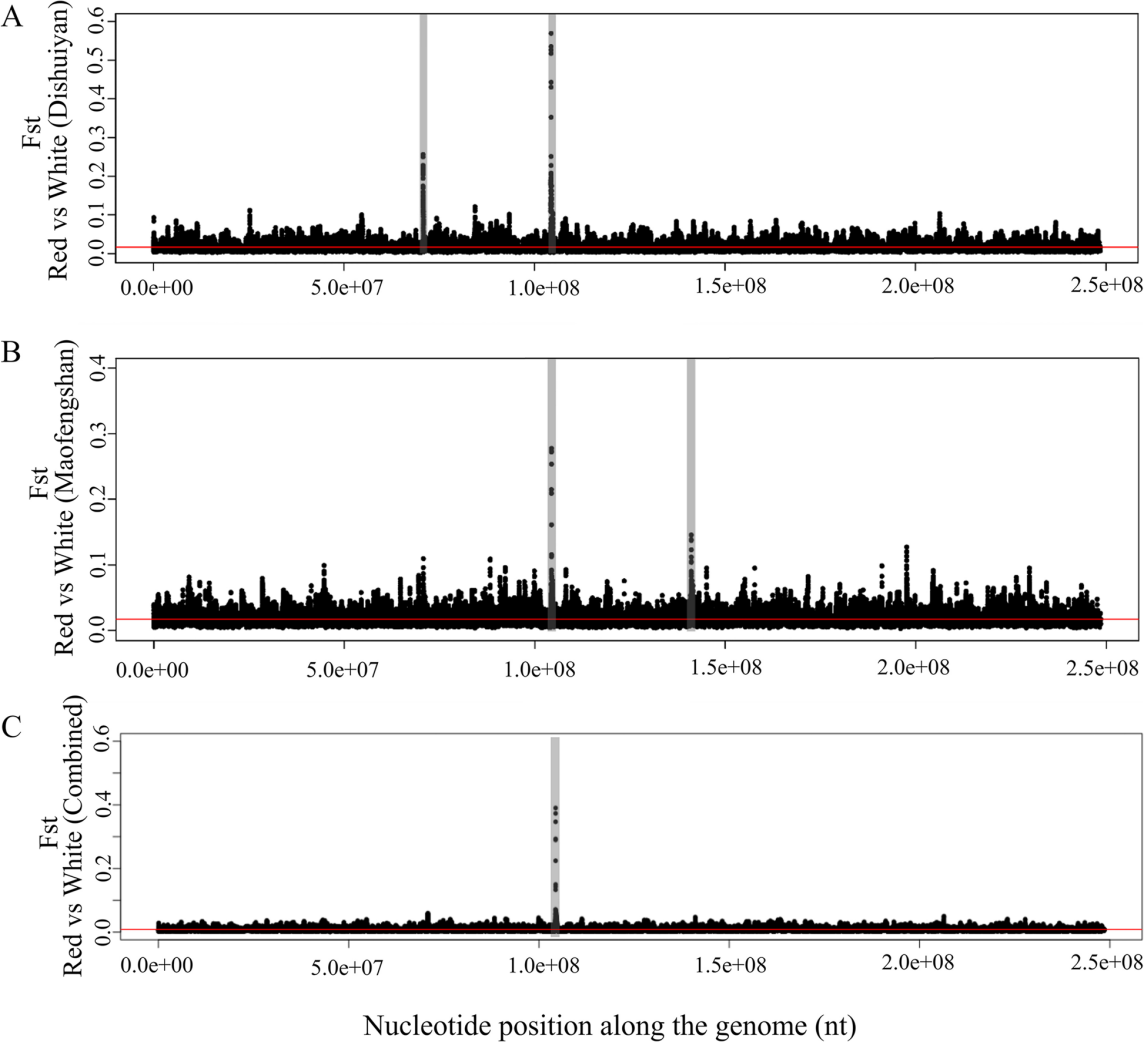
Genetic differentiation (Fst) between the Red morph and White morph of *Melastoma normale*. This is based on a sliding window analysis of Pool-seq data. Each dot represents the Fst of a 5-kb window. The average Fst across the genome was marked by a red line. Panels **A** and **B** show genetic differentiation (Fst) between the Red morph and White morph in Dishuiyan and Maofengshan, respectively, and **C** shows genetic differentiation (Fst) between the Red morph and White morph using the combined data from the two locations

A very low level of genetic differentiation was detected between the White and Red morphs from both sampling locations (Fig. 3A and B). The average Fst values between the two morphs in Maofengshan and Dishuiyan were 0.017 and 0.016 (Additional file 1: Table S4), respectively, suggesting substantial gene flow between the two morphs in each of the sampling locations. In contrast, significantly higher genetic differentiation (Fst = 0.064 for both cases) were observed between the same morphs sampled from Maofengshan and Dishuiyan (Wilcoxon rank sum test, *P* < 2.2 × 10^−16^; Additional file 1: Fig. S4), suggesting that geographic isolation might play a role in restricting gene flow.

Although the average genetic differentiation between the two morphs in both sampling locations was very low, in each sampling location, two highly differentiated genomic regions with the highest Fst values were observed (Fig. 3A, B). One of these genomic regions was shared between the two sampling locations, while the other genomic region was private for each sampling location. When using combined sequence data of each morph from the two sampling locations for analysis, only one genomic region showing very high genetic differentiation was detected between the two morphs compared with a very low level of genomic differentiation (Fig. 3C). This region was the same as the shared highly differentiated region mentioned above. This analysis suggests that other highly differentiated regions between the two morphs observed in each location are location-specific and may contain modifier loci of the major locus or simply represent spurious peaks in divergence. This highly differentiated genomic region was then considered as the candidate locus. It corresponds to a region on scaffold27 of the reference genome of *M. candidum*, where high-density SNPs with much elevated Fst values within a 6-kb interval (503000–509000) were found. Site by site Fst values across the genome were also calculated between the two morphs in each sampling location and all 50 SNPs with the highest Fst values (0.640 < Fst < 0.916) across the genome from Dishuiyan, and 46 of 50 SNPs with the highest Fst values (0.367 < Fst < 0.668) from Maofengshan were located in this interval (Additional file 1: Tables S5 and S6). The reliability of high-level differentiation at this region between the White and Red morphs in *M. normale* was verified using Sanger sequencing of all 109 individuals used for Pool-seq. The genic region of *myb114* (~ 1 kb in length), the only gene annotated in this 6-kb region (see below), was then the focus of subsequent analysis. High-level differentiation at this gene was confirmed between the two morphs from both sampling locations (Additional file 1: Table S7). Moreover, low genetic differentiation at this gene between the same morphs from the two sampling locations was also consistent with the Pool-seq analysis.

By searching genomic annotation of the reference genome of *M. candidum*, only one annotated gene (Mc_07625), which encodes a R2R3 MYB transcription factor in the 6-kb interval, was found. This R2R3 MYB transcription factor of *M. candidum* has homology to the MYB114/MYB75 subfamily in *Arabidopsis thaliana*, with 64% identity in amino acid sequence. It was designated as *myb114* hereafter. Gene annotation showed that *myb114* of *M. candidum* is 1059 bp in length from the start codon to the stop codon (scaffold27: 505966–507024), containing three exons and two introns. Along with the known roles of *myb114* in regulating gene expression of anthocyanin biosynthesis pathway in plants [43], *myb114* should be a major gene associated with the twig trichome color variation in *M. normale* (see haplogroup and twig trichome color association analysis below).

### Two copies of *myb114* were detected in *M. candidum* but only one in *M. normale*

**Fig. 4:**
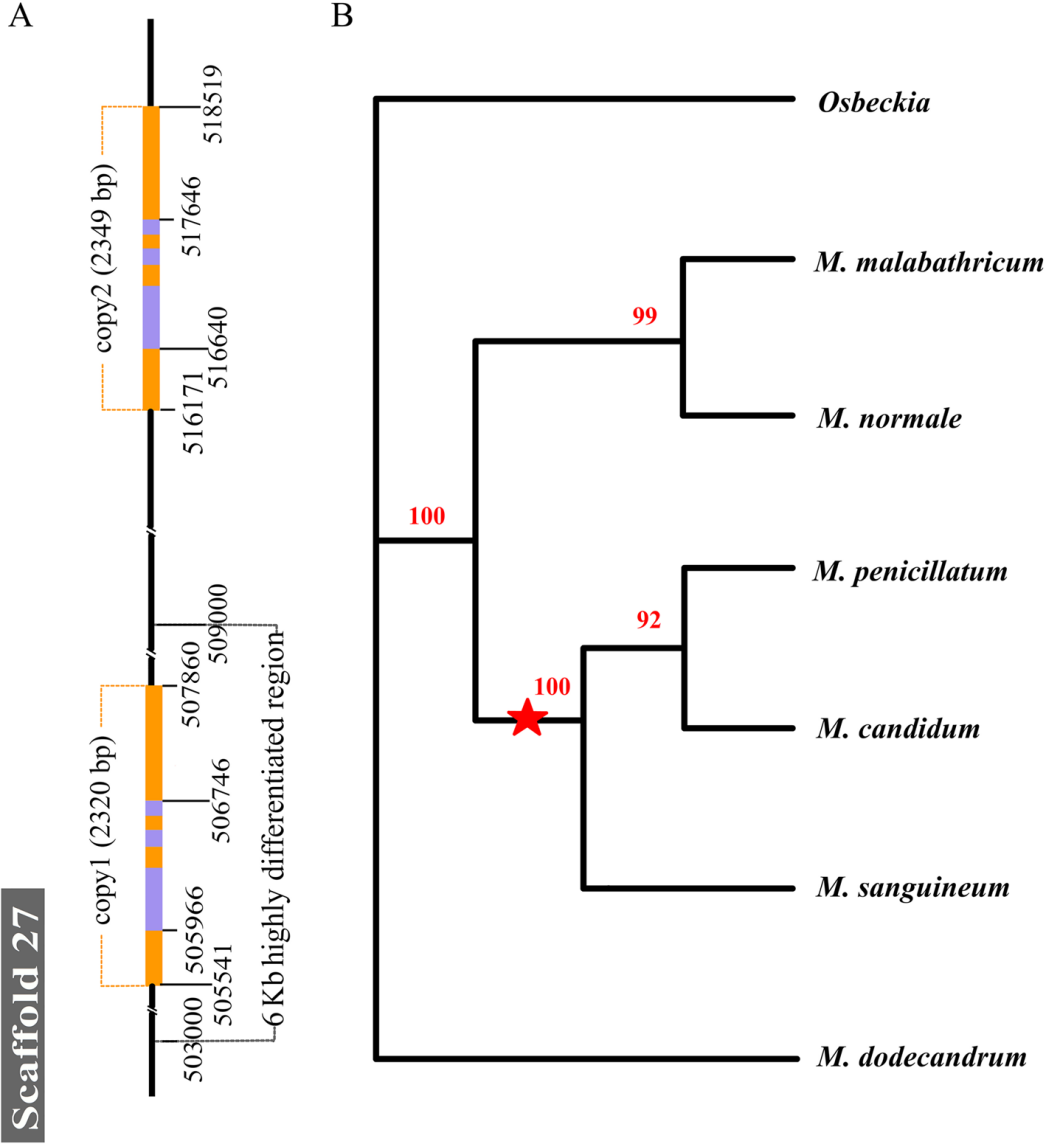
Duplication of the regions containing *myb114* in *Melastoma*. **A** Duplication of the regions containing *myb114* on scaffold27 of *Melastoma candidum*. The duplicate regions (copy 1 and copy 2 in yellow) are 2.3 kb long and 10 kb away from each other. The 6-kb highly differentiated region in *M. normale* corresponds to the left box with a black dotted line. The purple boxes in the duplicate regions represent exons of the *myb114* genes. **B** A schematic diagram showing evolutionary events occurring in *myb114* of *Melastoma* species. The phylogenetic tree was constructed based on chloroplast genome sequences of these species. The red triangle represents the occurrence of the 2.3-kb duplication event, which produced copy1 and copy2 regions in the common ancestor of *M. candidum, M. sanguineum*, and *M. penicillatum*

To test whether closely related paralogs exist in the genomes of *M. candidum* and *M. normale* as this can cause errors in read mapping and thus Fst calculation, the reference genome of *M. candidum* was first searched using Blast with the 6-kb sequence as a query and a 2.3-kb area (scaffold27: 516172–518520) having a 96% identity with an area (scaffold27: 505542–507861) was found within the 6-kb interval. In *M. candidum*, this 2.3-kb area is about 10 kb away from the 6-kb region and also contains an annotated R2R3 MYB transcription factor gene (Mc_07626) (Fig. 4A). A Blast search against the draft genome assembly of *M. normale* just available very recently was then done and only one area of 6.3 kb in pseudochromosome8 of *M. normale* (pseudochromosome8: 4658357–4664644), which has a 96% identity with the 6-kb interval, was found. This strongly suggests that there has been either a gene duplication in *M. candidum* or a gene deletion in *M. normale*. These two areas were called as copy1 and copy2 and, as each area contains a *myb114* gene, the two genes were denoted as *myb114-1* and *myb114-2* in *M. candidum*. Genome resequencing of one individual each of *M. candidum* and *M. normale* showed that read depth of copy1 and copy2 in *M. candidum* was similar to the genome average, while in *M. normale*, read depth of copy1 is similar to the genome average, but that of copy2 is zero or extremely low at most nucleotide sites (Additional file 1: Fig. S5). Read depth of copy1 and copy2 in the Pool-seq data of *M. normale* was very similar to that in the *M. normale* individual (Additional file 1: Fig. S5). PCR amplification and Sanger sequencing showed that most of the copy2 region in *M. candidum* has no corresponding sequence in *M. normale* due to the deletion of a 6-kb region in the latter (Additional file 1: SI text 1). Thus, unlike *M. candidum* which has two highly similar copies of *myb114* in the genome, *M. normale* has only one copy, *myb114-1* (*myb114* for short in *M. normale* hereafter since it has only one copy). In conjunction with the *myb114* copy number information in other species of *Melastoma* (two copies in *M. candidum, M. sanguineum*, and *M. penicillatum*, and one copy in *M. dodecandrum* and *M. malabathricum*; SI text 1) and chloroplast genome-based phylogeny of these species (Fig. 4B), the common ancestor of *Melastoma* appears to have a single copy of *myb114* and gene duplication of *myb114* occurred later in the common ancestor of *M. candidum*, *M. sanguineum*, and *M. penicillatum*. Moreover, sequence divergence between *myb114-1* and *myb114-2* of *M. candidum* (Ks = 0.0591; Kimura-two-parameter distance (*d*) = 0.0247) is much lower than that of *myb114-1* between *M. candidum* and *M. dodecandrum* (Ks = 0.1117; *d* = 0.0385), also supporting the duplication of *myb114* after divergence from *M. dodecandrum*.

### Two haplotype groups of *myb114* are highly divergent and geographically widespread in *M. normale*

**Fig. 5:**
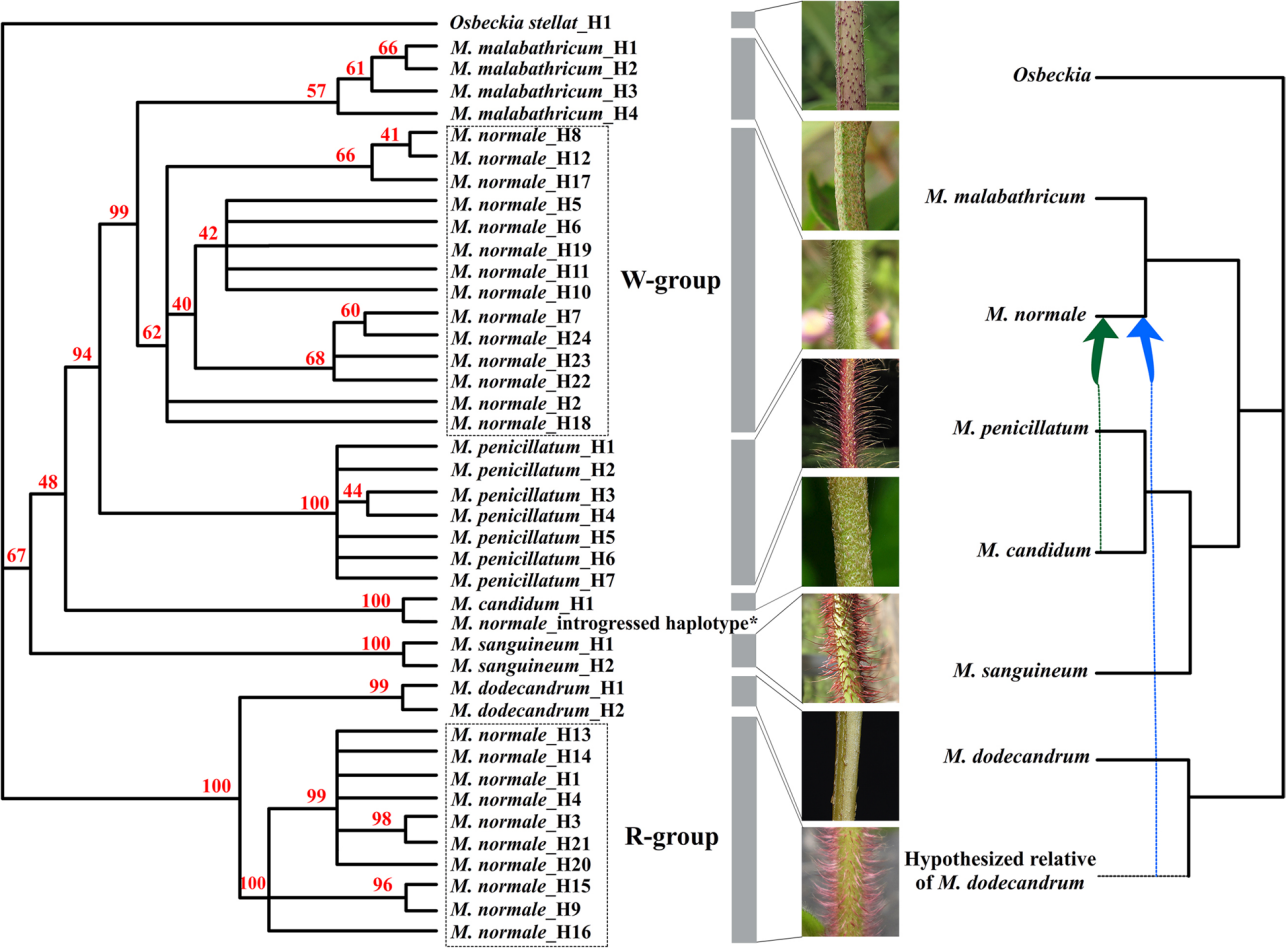
Phylogenetic analysis of the *myb114* genes of six species of *Melastoma* in China. The tree was constructed using the maximum parsimony algorithm*.* The twig trichome trait of each species was shown with a photo. The W-group and R-group haplotypes of *M. normale* are indicated in the boxes with dotted lines. Nodes with bootstrap value less than 40% are collapsed. * indicates the haplotype introgressed from *M. candidum*. The *myb114* introgression events are shown on the chloroplast genome tree of these species, with the blue dotted arrow representing introgression from an unsampled/extinct relative of *M. dodecandrum* to *M. normale*, and the green dotted arrow representing very recent or ongoing introgression from *M. candidum* to *M. normale*

Sanger sequencing of *myb114* in all 109 individuals used for Pool-seq showed that there was introgression from *M. candidum* in 17 individuals of *M. normale* because one of the two haplotypes for each of these individuals had the same sequence as *M. candidum* after clonal sequencing (Fig. 5). *M. candidum* has brown appressed scales on its twigs and it seems that introgression from *M. candidum* has little influence on this trait of the 17 individuals of *M. normale*. Introgression from *M. candidum* is not surprising because *M. normale* coexists with *M. candidum* in the two sampling locations and many other regions in South China. Although *M. normale* flowers primarily from March to April in Guangzhou, much earlier than *M. candidum* which flowers in June to August, occasional secondary flowering in *M. normale* may cause hybridization and introgression. The introgression explanation was also supported by sequence analysis in an allopatric *M. normale* population sampled from Zigong, Sichuan, where no other species of *Melastoma* exist and no haplotypes of *M. candidum* were detected. The individuals possessing haplotypes introgressed from *M. candidum* were removed for later analysis.

**Fig. 6:**
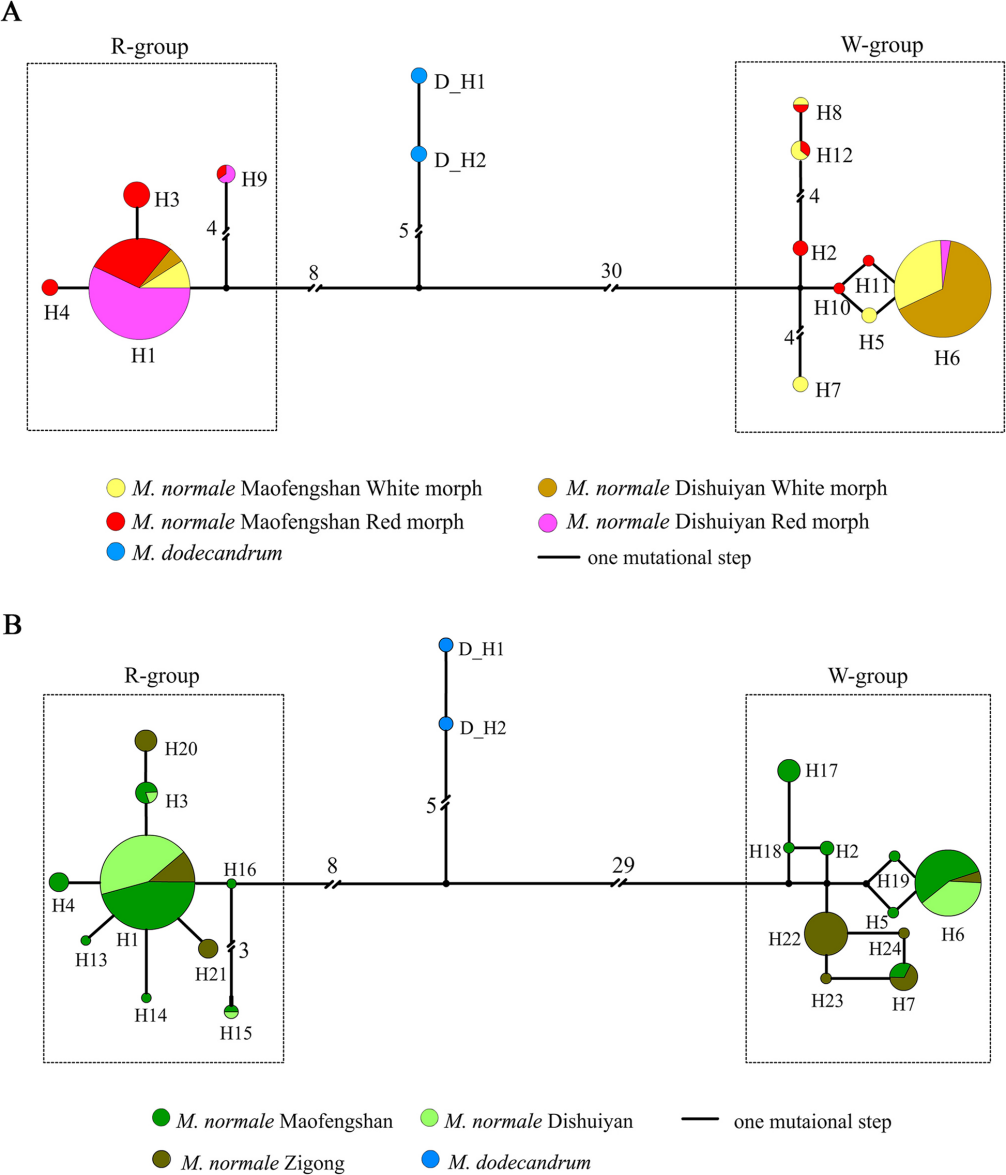
Haplotype network of the *myb114* gene of *Melastoma normale*. Panel **A** is the haplotype network for the samples used for Pool-seq and panel **B** is that of three randomly sampled populations. Two haplotypes of *M. dodecandrum* were added into the network as outgroups. Haplotypes of each population are shown in one color. The sizes of the circles correspond to frequencies of the haplotypes. Small black circles represent hypothetical haplotypes. Mutational steps are shown by the number near the connecting lines, with the number omitted for those with only one or two mutation steps. See the main text for the definition of the R-group and W-group. The same codes were used for shared haplotype between panels **A** and **B**

Haplotype analysis of *myb114* in *M. normale* revealed two highly divergent haplogroups with 37 differentially fixed nucleotide substitutions and indels between them (Fig. 6A). The two haplogroups were denoted as W-group (haplotypes in this group are predominantly from the White morph) and R-group (haplotypes in this group are predominantly from the Red morph). The haplogroups showed strong association with the twig trichome color (Additional file 1: Table S8). The most frequent haplotypes within the W- and R-groups are H6 and H1, respectively (Fig. 6A). The frequencies of H6 in the White morph of the two sampling locations are both higher than 0.6, while those of H1 are lower than 0.2 (Additional file 1: Table S9). By contrast, the frequencies of H1 in the Red morph of the two sampling locations are both higher than 0.6, while those of H6 are both lower than 0.1 (Additional file 1: Table S9). At the amino acid level, there were 14 amino acid (aa) substitutions and two 1-aa indels between H1 and H6. Notably, no WW genotypes (both alleles are from the W-group) in the sampled individuals of the Red morph were found in both Dishuiyan and Maofengshan, and no RR genotypes (both alleles are from the R-group) in the sampled individuals of the White morph were observed in Dishuiyan. However, three individuals of the White morph in Maofengshan were found to have RR genotypes (H1H1 for all three individuals). It was speculated that recent loss-of-function mutation in the regulatory region of *myb114* or in other gene(s) interacting with it causes the failure of anthocyanin synthesis in their twig trichomes.

To exclude the potential influence of non-random sampling in the Pool-seq analysis and to conduct the following population genetics analysis, the *myb114* gene of 50 randomly sampled individuals each from Maofengshan and Dishuiyan, regardless of the twig trichome color, was sequenced. Again, the individuals possessing haplotypes introgressed from *M. candidum* were removed from later analysis. Because this introgression event is not associated with the twig trichome variation in *M. normale*, it will not be mentioned hereafter to avoid confusion. Fourteen haplotypes of *M. normale* from the two sampling locations were detected (Fig. 6B), and again, they formed two highly divergent haplogroups, W-group and R-group, as mentioned above. For each haplogroup, the same major haplotypes, that is, H6 in the W-group and H1 in the R-group, were found. The two highly divergent haplogroups at a considerable and similar frequency were also detected in a population sampled from Jianshan, Zigong, Sichuan, which is more than 1000 km away from Guangzhou (Fig. 5B). Therefore, the two highly divergent haplogroups should be geographically widespread in *M. normale*.

The high level of sequence divergence among the two haplogroups suggests that they have coexisted for a long time to build up genetic divergence or that one of the haplogroups was introgressed from other species. To infer how the two highly divergent haplogroups of this gene in *M. normale* formed, phylogenetic relationships of six species of *Melastoma* from China were reconstructed based on haplotype sequences of this gene. Haplotypes of *M. normale* were from 50 randomly sampled individuals each from Maofengshan and Dishuiyan, and 20 individuals from Jianshan. Haplotypes of other species were from five individuals for each species. Because some species have two copies of *myb114*, *myb114-1*-specific primers were designed for PCR amplification. As shown in the maximum parsimony tree (Fig. 5), which had some differences from the chloroplast genome tree, W-group haplotypes of *M. normale* were sister to those of *M. malabathricum*, consistent with their close relationships (The two species were once treated as two subspecies of the same species [36]), while the R-group haplotypes of *M. normale* were closely related to those of *M. dodecandrum*, the mostly diverged species in *Melastoma*. This suggests that the highly divergent R-group haplotypes may have originated from introgression from *M. dodecandrum* or other unsampled/extinct species closely related to *M. dodecandrum*. Considering that *M. dodecandrum* has no red, dense and spreading twig trichomes, introgression from other unsampled/extinct species closely related to *M. dodecandrum* seems more likely. This introgression event is unlikely to be very recent, given the observations of geographically widespread range of this variation and a short, highly differentiated region (6 kb) between the two morphs.

### Balancing selection maintains the two highly divergent haplogroups in *M. normale*

The geographically widespread, strong divergence at the 6-kb region contrasts with the extremely low genetic differentiation across the genome between the Red and White morphs of *M. normale*, suggesting that selection may maintain this divergence despite populations of the two morphs experiencing substantial homogenizing gene flow. An alternative explanation is that this strong divergence at the 6-kb region resulted from only neutral introgression. To determine which explanation holds in this case, the following analyses were conducted.

First, a population genetic analysis was done on the sequence data of *myb114* from 50 randomly sampled individuals each from Maofengshan and Dishuiyan. At the *myb114* gene, Tajima’s D were significantly larger than 0 for both Maofengshan (D = 2.82, *P* < 0.01) and Dishuiyan (D = 2.08, *P* < 0.05) populations. Site frequency spectrum analysis also showed that there were excessive moderate and even high-frequency mutations but much fewer low-frequency mutations in both populations, remarkably different from the expected distributions under neutrality (Additional file 1: Fig. S6). The nucleotide diversity at this gene in Maofengshan and Dishuiyan populations was 0.018 and 0.014, respectively, much higher than the genomic average (0.008–0.009). In addition, the level of linkage disequilibrium (*r*^2^) between segregating sites at the *myb114* gene was measured. *r*^2^ is close to 1 for many sites throughout the *myb114* gene, suggesting that most nucleotide sites are completely correlated (Additional file 1: Fig. S7). No recombination between the two highly divergent haplogroups and within each haplogroup was detected in the Dishuiyan population, and in the Maofengshan population, evidence of recombination was found only within the W-group, with the minimum number of recombination events being eight. Hence, the absence of recombination between the two haplogroups is not due to a reduction in crossovers in this region. Instead, recombination appears to be suppressed between haplogroups, but not within. All these results are in line with balancing selection, which can result in an excess of intermediate-frequency variants in the site frequency spectrum, increased diversity [4] and elevated levels of LD around the target of balancing selection [44–46]. Although severe population shrinkage can also cause an excess of intermediate-frequency variants and elevated levels of LD, that the genomic average of Tajima’s D is lower than 0 (Additional file 1: Fig. S3) suggests that it is unlikely. However, these patterns are also consistent with the explanation of neutral introgression if the introgressed alleles are at a considerable frequency in these populations.

Second, two recently developed, more powerful and robust summary statistics, β^(2)^ and Non-central Deviation (NCD2) [20, 21, 47], were used to scan the genomes of each of the three populations for signatures of balancing selection. The results showed that *myb114* had significantly stronger signatures of balancing selection than the whole genome in all three populations whether using *β*^(2)^ or NCD2 (Table 1; Additional file 1: Fig. S8). Mean *β*^(2)^ scores at *myb114* were 24.98, 16.41 and 12.03 in Jianshan, Dishuiyan, and Maofengshan populations, respectively, significantly greater than the genome-wide averages, which were 7.72, 6.55, and 6.53, respectively. When using NCD2, there was a significant reduction in NCD2 scores (indicating stronger signatures of balancing selection) in all three populations.

**Table 1:** Signatures of balancing selection in *myb114* in three populations of *Melastoma normale*. This is detected for the *myb114* gene relative to the whole genome using the β^(2)^ and NCD2 scores. *N* in the third and fourth columns refers to the number of SNPs used in the comparison. SE refers to standard error. One-sided Wilcoxon rank sum tests were performed in the R package

**Quantity tested**	**Population**	**SNPs of myb114 mean ± SE (N)**	**SNPs of the whole genome mean ± SE (*N*)**	***P* value**
**β**^**(2)**^** score**	Dishuiyan	16.41 ± 1.70 (29)	6.55 ± 0.0050 (2105786)	1.15E−07
	Maofengshan	12.03 ± 0.69 (47)	6.53 ± 0.0043 (2580849)	1.08E−11
	Jianshan	24.98 ± 1.46 (26)	7.72 ± 0.0071 (2213379)	2.09E−12
**NCD2 score**	Dishuiyan	0.25 ± 0.0025 (29)	0.35 ± 0.000038 (2105786)	2.20E−16
	Maofengshan	0.33 ± 0.00082 (47)	0.36 ± 0.000029 (2580849)	1.33E−09
	Jianshan	0.20 ± 0.0066 (26)	0.35 ± 0.000046 (2213379)	2.20E−16

In a complementary manner, 15, 11, and 4 SNPs at *myb114* were in the top 5% of the *β*^(2)^ distributions in Jianshan, Dishuiyan, and Maofengshan populations, respectively, and 6 and17 SNPs at *myb114* were in the bottom 5% of the NCD2 distributions in Jianshan and Dishuiyan populations, respectively. No SNPs at *myb114* were in the bottom 5% of the NCD2 distributions in Maofengshan population, and 4 SNPs with the lowest NCD2 scores were in the bottom 20% of the NCD2 distributions, which might be caused by more introgression from *M. candidum* in this population.

Third, the frequency of the introgressed alleles in each population was calculated. For this analysis, the Jianshan population in which no other *Melastoma* species exist was also included. Based on the results mentioned above, alleles from the R-group stem from introgression. The frequencies of the introgressed alleles in Maofengshan, Dishuiyan, and Zigong populations are 0.622, 0.714, and 0.719, respectively. Because the frequency of introgressed alleles is expected to decrease due to the continuous backcross to the recipient species (*M. normale* in this case), the high frequency of introgressed alleles is unusual if they are not favored by selection. In this case, the frequency of introgressed alleles is expected to decrease more because the donor species cannot be found in China or even may be extinct. At least for the population from Jianshan, the highest frequency of the introgressed alleles is opposite to the expectation under neutral introgression.

Fourth, the nucleotide diversity and polymorphism of *myb114* versus surrounding regions were compared. For this comparison, the individuals of *M. normale* from Maofengshan population were classified according to *myb114* genotype. There are 14 individuals with RR genotype (both alleles are from the R-group), and 12 with WW (both alleles are from the W-group). Six single-copy segments (800–900 bp per segment) distributed across a ~60-kb region centered on *myb114* were sequenced in the 26 individuals. For the individuals with RR genotype, the nucleotide diversity and polymorphism of the six surrounding segments ranges from 0.00334 to 0.00811, and from 0.00269 to 0.00951, respectively; for the individuals with WW genotype, the nucleotide diversity and polymorphism of the six surrounding segments ranges from 0.00307 to 0.00863, and from 0.00419 to 0.00892, respectively (Additional file 1: Table S10, Fig. S9). The nucleotide diversity and polymorphism of the six surrounding segments are significantly higher than those of *myb114*, which are 0.000470 and 0.00123 for the individuals with RR genotype, and 0.00229 and 0.00300 for the individuals with WW genotype, respectively (Additional file 1: Fig. S9, Table S11). The trend is especially obvious for the individuals with RR genotype. For both RR- and WW- genotype bearing samples, the low diversity at *myb114* relative to surrounding regions suggests that both R and W alleles exhibit a molecular signature of positive selection rather than being neutral. Taken as a whole, *myb114* should be under balancing selection in *M. normale*.

To further investigate the mechanisms of balancing selection acting on this gene, these individuals were classified as homozygotes or heterozygotes of the two haplogroups “R” and “W”. Based on sequence data of *myb114* in Maofengshan and Dishuiyan populations, genotype frequencies of WW, RR, and WR (one allele from the W-group and the other from the R-group) were obtained. Although both populations have a considerable fraction of WR genotype (0.593 and 0.561 in Maofengshan and Dishuiyan populations, respectively), no significant deviation from Hardy-Weinberg equilibrium was detected (*X*^2^ = 1.526, *p* > 0.05 for Maofengshan population; *X*^2^ = 1.395, *p* > 0.05 for Dishuiyan population). This indicates that heterozygote advantage may not be the mechanism of balancing selection on *myb114* in *M. normale*. Instead, considering that there is substantial variation in light intensity within most populations of *M. normale*, for example, from open roadsides with full sunlight to understory habitats with much less sunlight, the two morphs appear to differentially adapt to habitats with high and low sunlight intensity and that both R and W alleles of *myb114* have been under positive selection, spatially varying selection is a more likely mechanism of balancing selection in this case.

## Discussion

The geographically widespread twig color variation within populations of *M. normale* and the availability of a reference genome facilitates genetic dissection of this trait. More interestingly, the common garden experiment indicates that the Red and White morphs display differential adaptation to heterogeneous microhabitats. With available reference genomes, Pool-seq has the advantage of efficiently identifying the genomic regions associated with phenotypic variations [48]. Using this approach, the twig trichome color variation in *M. normale* is found to be largely associated with a R2R3 MYB transcription factor, which is highly divergent between the two morphs despite a very low level of background genomic differentiation. MYB transcription factor gene family has a large number of members in flowering plants, with important regulatory roles in plant development and responses to biotic and abiotic stress [49, 50]. Among MYB genes, the two-domain R2R3 MYB genes often exhibit tissue-specific patterns of expression and have been implicated in trichome development (e.g., [14, 51]) and pigmentation variation in many tissues of plants (e.g., [52–55]). For example, an R2R3 MYB gene of the MYB48/MYB59 subfamily is largely associated with leaf trichome density divergence (glabrous versus densely hairy) in *Mimulus guttatus* [14]. The members in the MYB114/MYB75 subfamily (including MYB75, MYB90, MYB113, and MYB114) can regulate the anthocyanin biosynthesis genes later in the pathway, leading to the synthesis of pro-anthocyanidins and anthocyanins [43, 56]. To date, no studies have found any functional connection of R2R3 MYB transcription factors to trichome color in other plants. This study indicates that R2R3 MYB transcription factors are also implicated in trichome pigmentation variation. Further work is required to figure out whether the coding or *cis*-regulatory regions of *myb114* carry the causal mutations. However, the large number of amino acid differences differentially fixed in the two haplogroups makes it difficult to point to any single amino acid change as the causal mutations. It seems likely that the two groups of alleles differ functionally, perhaps reinforced by additional differences in the *cis*-regulatory region.

In *M. normale*, twig trichome color variation appears to be associated with a locus of large effect. A similar genetic basis has also been found for pigment trait divergence of multiple plants and animals (e.g., [12, 57–60]). It is expected that single-locus architecture or tight linkage between the loci involved (or a supergene) is responsible for traits under balancing selection [61]. Yeaman and Whitlock also predicted that genetic architecture of local adaptation in the face of gene flow should be due to few large-effect loci or multiple tightly linked small-effect loci [42]. Previous studies [12, 14] and this study are consistent with these predictions.

Phylogenetic analyses indicate that one of the two highly divergent haplogroups at *myb114* in *M. normale* arose from introgression from unsampled/extinct species closely related to *M. dodecandrum*, and that the introgression event is not very recent and the forces of balancing selection should be strong and continuous over time to prevent the loss of this polymorphism. Introgression from related species is an important mode of adaptation (e.g., [62, 63]). The long-term persistence of polymorphisms has also been seen in other species, such as *Drosophila* [46], ruff [64] and Gouldian finch [60]. The number of segregating sites, haplotype diversity, and nucleotide diversity in the R-group are all lower than those in the W-group, which may be caused by stronger selection on this haplogroup. However, this pattern is also consistent with there being multiple knock-out mutations in the regulatory regions possible to create the white phenotype but only one way to have a functional red copy.

Interspecific hybridization is common in *Melastoma* (e.g., [65–67]). However, except for *M. normale*, no any other instances of introgression of the R-group haplotypes into *Melastoma* species in China have been observed. Different adaptive traits or different ecological requirements in other species may be the main reason. For *M. candidum* and *M. malabathricum*, overlapping appressed scales on their twigs may offer effective protection for twigs from intense sunlight. For *M. sanguineum* and *M. penicillatum* with their twigs being covered by hard and soft bristles, respectively, they occur in slightly shady or shady habitats, where light intensity is relatively weak. Considering that the number of species of *Melastoma* in China takes up only 1/10 of the whole genus, it is possible that the R-group haplotypes of *myb114* might have introgressed into some species in Southeast Asia. This is an interesting venue for future investigation.

A very high level of linkage disequilibrium (LD) at the *myb114* gene was observed in each population of *M. normale*. Increased LD in this gene indicates that effective recombination was reduced in this genomic region. This is consistent with balancing selection acting on this gene in these populations. Alternatively, this can also be caused by a selectively neutral inversion polymorphism. However, no signal of inversion in the 6-kb region was detected after checking read mapping of the two Pool-seq samples from Dishuiyan. Unlike many other cases caused by recent selective sweeps, in which high differentiation can extend over larger genomic regions, the region of high differentiation between the two morphs of *M. normale* is only about 6 kb. This is unsurprising because LD is expected to decay with increasing physical distance from single selected locus over tens to thousands of generations by recombination [68–70]. Balancing selection on *myb114* over a relatively long time in *M. normale* and short generation time of *Melastoma* species (two years) can account for this. A small, highly differentiated region is consistent with the observations in humans, where signatures of long-term balancing selection are confined to regions of at most a few kilobases [71].

Elucidating the selective mechanisms for maintaining variations in natural populations has been a fundamental topic in evolutionary biology. Population genetics analyses in this study suggest that balancing selection could contribute to maintaining the twig trichome color variation in *M. normale*. Divergent natural selection occurring between populations (or ecotypes) in different habitats is an important cause of phenotypic variation in species [72–74]. Microhabitat heterogeneity and divergent natural selection, that is, spatially varying selection, likely supports the maintenance of the two morphs of *M. normale*. The heterogeneous microhabitats with high and relatively low sunlight intensity can have opposing selective advantages for the Red and White morphs, respectively. Individuals of the Red morph may have a selective advantage in open habitats as red twig trichomes may resist strong UV radiation to protect twigs. Anthocyanins play a key role as “light filters” against high light stress (especially UV) in protecting the photosynthetic machinery and thus preventing photoinhibition [75, 76]. Individuals of the White morph may have a selective advantage in slightly shady habitats as it will not invest additional energy to synthesize anthocyanins in twig trichomes to ensure more investment on growth and reproduction. Anthocyanin production is an energy-demanding process and therefore strong selection against its accumulation in relatively low light intensity is expected.

## Conclusions

The main findings in this study include as follows: (1) Twig trichome coloration in *M. normale* is under selection in different light environments; (2) An R2R3 MYB transcription factor gene is the major locus associated with twig trichome color variation in *M. normale*; (3) This transcription factor gene has two highly divergent groups of alleles, one of which likely originated from introgression from another species of this genus; (4) This gene is under balancing selection and spatially varying selection is the most likely mechanism of balancing selection. Moreover, this study also explains how adaptive divergence can occur and be maintained in the face of gene flow.

## Methods

### Common garden experiments

One ripe fruit (several hundred seeds per fruit) was randomly collected from each of 20 individuals of *M. normale* from Jianshan, Zigong, Sichuan. These individuals were randomly selected regardless of their twig trichome colors. The seeds were harvested, pooled, and sowed in a plastic tray filled with the peat soil mix 933 (Klasmann-Deilmann, Germany). After 3 months, 60 seedlings with the extreme red twig trichomes and 60 seedlings with the extreme white twig trichomes were screened by eye. Three-month-old seedlings of *M. normale* were planted in 10 cm × 10 cm plastic pots filled with the same soil, with one seedling in each pot. For each morph, 30 seedlings each were grown in the full sunlight (high light intensity) and a sunshade net with a shading rate of 40% (low light intensity) in the greenhouse of Sun Yat-sen University. The temperature in the greenhouse was 30 °C during the day and 20 °C during the night. The plants were watered to maintain soil moisture at about 70%. After 9 months of growth, all plants (aboveground biomass) were harvested and then dried at 75 ℃ in an oven for 48 h to measure their dry weight. Above ground biomass was used to represent the fitness because the lifetime fitness could not be measured, given that the life time of this species is more than 10 years. A two-way ANOVA test was performed to analyze the effect of morph and sunlight intensity on dry weight using SPSS UNIANOVA. Because there is a significant interaction between morph and sunlight intensity, pairwise significant differences in plant dry weight at high and low light intensities for the two morphs were determined with LSD tests using the same subprogram UNIANOVA.

### Plant sampling

For Pool-seq, the individuals of *M. normale* were sampled from two locations in Guangzhou: Maofengshan Forest Park in Baiyun District, and Dishuiyan Forest Park in Panyu District. For short, the two locations were named as Maofengshan and Dishuiyan hereafter. Geographical distance between them is about 70 km. For each location, 150 individuals were randomly sampled regardless of their twig trichome colors. One branch of each individual was collected. When these fresh materials were taken to the lab, 40 individuals with the extreme twig trichome colors (red and white) were first screened by eye for each morph from each location, and of the 80 individuals 24 and 25 individuals with the extreme twig trichome colors (red and white) from Maofengshan, respectively, and 30 and 30 from Dishuiyan, respectively, were then selected with a stereo microscope.

For population genetics analyses of the *myb114* gene, 50 individuals of *M. normale* each from Maofengshan and Dishuiyan, and 20 individuals from a distant population from Jianshan, Zigong, Sichuan, were also randomly sampled regardless of the twig trichome color. Fifteen of the 20 individuals of *M. normale* were used for genome resequencing to calculate β^(2)^ and NCD2 described below. To characterize the copy number of the identified highly differentiated region in *M. candidum*, *M. normale*, and *M. dodecandrum*, one individual each of the three species was also sampled from Wenchang in Hainan, Guangzhou, and Nanping in Fujian, respectively, which were used for genome resequencing. In addition, five individuals each of four other species of *Melastoma* in China were sampled for phylogenetic analysis of the *myb114* gene.

Leaves of all samples mentioned above were collected and dried with silica gels. The sampling details were shown in Additional file 1: Table S1.

### DNA isolation

The CTAB method [77] was used for DNA isolation. For Pool-seq samples, the dried leaf tissues of all the individuals were equally pooled, as conducted by Zhou et al. [78], after screening by the stereo microscope for each morph from each location. DNA of each individual used for Pool-seq was also isolated for subsequent validation experiment. For other samples, DNA was isolated for each individual separately.

### Illumina sequencing

Genomic DNA libraries with 400 bp insert size were constructed for the four pooled samples of *M. normale*, one sample each of *M. candidum* and *M. dodecandrum*, and 15 of 20 individuals of *M. normale* sampled from Jianshan using the Illumina TruSeq Library Preparation Kit following the manufacturer’s protocol. These libraries were sequenced on an Illumina Hiseq X10 platform in Berry Genomics, Beijing, and more than 30 Gbp paired end (150-bp) reads were obtained for each library. About 8 Gbp reads were obtained for one sample each of *M. candidum*, *M. normale*, and *M. dodecandrum*. All these short reads were deposited in GenBank with accession numbers SRR8892966-SRR8892971 and SRR19183013-SRR19183030.

### PCR amplification and Sanger sequencing

In this study, PCR amplification and Sanger sequencing were conducted for multiple purposes: (1) to verify the highly differentiated region in *M. normale* identified by Pool-seq, (2) to determine the copy number of the *myb114* gene in *M. normale* and other *Melastoma* species, (3) to carry out population genetics analyses of *myb114* and six surrounding segments in *M. normale*, and (4) to reconstruct the phylogenetic tree of *Melastoma* species in China based on sequences of the *myb114* gene. All the primers were designed based on the reference genome of *M. candidum*, and primer sequences are listed in Table S12. PCR was conducted in a total volume of 25 µL with KOD FX DNA polymerase (TOYOBO, Osaka, Japan). The purified PCR amplification products were then directly sequenced on an ABI 3730 DNA automated sequencer with the BigDye chemistry (Applied Biosystems, Foster City, CA, USA). For sequences that contained multiple polymorphic sites, clonal sequencing was performed using the pMD-18 T Vector Kit (Takara, Dalian, China). Eight positive clones were sequenced to phase the haplotypes of each sample in DNASP. These sequences have been deposited in GenBank with the accession numbers MK618466-MK618508, MT010136-MT010215, and ON565433.

### Pool-seq data analysis

Trimmomatic version 0.33 [79] was used to trim adaptors and remove low-quality reads. The perl script IlluQC_PRLL.pl in the NGSQCToolkit version 2.3.3 [80] was further used to trim low-quality bases at both ends of the reads with the parameters -l 60 -s 13. FastUniq version 1.1 [81] with default parameters was used to remove PCR duplicates. The filtered reads of the four pooled samples were then separately mapped onto the *M. candidum* reference genome (GenBank accession number JARUPX000000000) [82] with BWA-MEM with default parameters (https://github.com/lh3/bwa). The filtered reads of the pooled samples of the same morph from the two locations were also combined and then mapped onto the *M. candidum* reference genome. The generated sam files were then converted to sort bam files through *samtools view* and *samtools sort* in Samtools version 1.4 [83]. Qualimap [84] was then used to check the mapping results of each sample onto the reference genome of *M. candidum*, including read depth and mapping quality. SNP information for each pooled sample and the two combined samples was extracted using Samtools *mpileup* with the minimal base quality 20 and minimal mapping quality 20. The pileup files of the four pooled samples and two combined samples were used for subsequent analyses.

POPOOLATION version 1.2.2 [85] was used to calculate nucleotide diversity (*π*), nucleotide polymorphism (*θ*_w_), and Tajima’s D for each morph from each of the two locations, Maofengshan and Dishuiyan. Indels were detected and removed for subsequent analysis using the perl scripts *identify-genomic-indel-regions.pl* and *filter-pileup-by-gtf.pl*. The new pileup file after indel filtering was used as input to calculate *π*, *θ*_w_, and Tajima’s D using *variance-sliding.pl*. Six parameters were set: (1) site sequencing coverage (1/2 × mean genomic coverage ≤ coverage ≤ 2 × mean genomic coverage); (2) minimum base quality (min-base-quality = 20); (3) pool size (pool size = the number of individuals used in the Pool-seq); (4) minimal minor-allele count (min-allele-count = 4); (5) sliding window size and step size (window size = 5 kb, step size = 1 kb); and (6) minimal fraction covering the window size (min-covered-fraction = 50%).

POPOOLATION2 version 1.201 [86] was further used to calculate genetic differentiation (Fst) between the two morphs. The parameters are the same as those mentioned above except for min-allele-count = 8 when the combined data were analyzed. Fst between the two morphs was also calculated using 10- and 20-kb sliding windows (step size = 2 and 4 kb, respectively) and similar results were obtained: the region containing *myb114* was always the highest differentiated one for all these window sizes (data not shown). Moreover, Fst was also calculated for each SNP site across the genome. To delimit the size of the highly differentiated region, Fst between the two morphs was calculated with a non-overlapping sliding window size of 1 kb. The size of the region was determined when the region contains consecutive windows with Fst values much higher than those of adjacent windows.

### Bioinformatics analysis for the highly differentiated region

Gene annotation information was searched based on the *gff3* file of the reference genome of *M. candidum*. Only one annotated gene, a R2R3 MYB transcription factor, was found within the 6 kb highly differentiated region. Blastp was further used to search against GenBank to identify the most similar gene in the Arabidopsis genome. To characterize the copy number of this region containing the *myb114* gene in the *M. candidum* genome, Local Blast in BioEdit v.7.1.3.0 [87] was used to search highly similar genomic regions. A 2.3-kb area, which is about 10 kb away from, and highly similar to, the highly differentiated region, was found. The two duplicates were called as copy1 and copy2. The copy2 area also contains an *myb114* gene. In addition, the copy number of *myb114* in *M. normale*, *M. sanguineum*, and *M. dodecandrum* was characterized by the same Blast analysis with their recently available genome sequences (GenBank accession numbers: JARUQA000000000, JARUPW000000000, and JARUPY000000000) [88–90].

To further determine if *M. normale* has the same two copies as *M. candidum*, the read depths of copy1 and copy2 areas were compared between *M. candidum* and *M. normale*. To detect potential structural variations (including large-fragment insertions, deletions, and inversions) in copy1 and copy2 areas relative to *M. candidum*, mapping information of read pairs with only one read successfully mapped onto the reference genome was checked from the sam files. Based on the mapping information, read pairs of this kind were extracted using Linux commands *grep* and *awk*, and then de novo assembled into contigs in SeqMan v.7.1.0 (Dnastar, Lasergene, Madison, WI, USA). Potential structural variation regions with specific PCR primers (Table S12) were then amplified and sequenced to identify structural variation and to see if *M. normale* has the same two copies as observed in *M. candidum*.

### Validation of the highly differentiated region identified by Pool-seq and association analysis between haplogroup and twig trichome color

To confirm the reliability of the highly differentiated region identified by Pool-seq, the *myb114* gene was amplified and then sequenced for all the individuals used in Pool-seq. PCR amplification and sequencing primers are shown in Table S12. DNA of each individual used for Pool-seq was separately amplified and sequenced. DNASP v. 5.10 [91] was used to calculate genetic differentiation (Fst) between the two morphs. The association between haplogroups and twig trichome colors was analyzed using Fisher’s exact test.

### Inference of the origin of the R-group haplotypes in *M. normale*

To figure out the origin of the R-group haplotypes in *M. normale*, the *myb114* gene was amplified and sequenced for five individuals each of five other species of *Melastoma* (*M. candidum*, *M. sanguineum*, *M. dodecandrum*, *M. malabathricum*, and *M. penicillatum*) in China. *Osbeckia stellata* was used as an outgroup because *Osbeckia* is sister to *Melastoma* [92]. Haplotype sequences of all species were aligned using MAFFT v.7.307 [93], and phylogenetic analysis was performed using maximum parsimony method in PAUP*v.4.0 [94]. The reliability of the phylogenetic tree was estimated by bootstrapping with 1000 replicates. Because *myb114* is under selection (see “Results” for details), the chloroplast genome sequences were used to reconstruct the species phylogeny using the same method. The chloroplast genome sequences of these species were downloaded from GenBank with accession numbers OQ595234-OQ595240 [95–101].

### Population genetics analysis of the *myb114* gene in *M. normale*

Fifty randomly sampled individuals of *M. normale* each from Maofengshan and Dishuiyan, and 20 individuals from a distant population in Zigong, Sichuan, were amplified and sequenced, regardless of twig trichome color. Again, clonal sequencing was used for haplotype phasing for samples with multiple heterozygous sites at this gene. Nucleotide diversity of each population was calculated in DnaSP. Tajima’s D test was conducted on the *myb114* gene for Maofengshan and Dishuiyan populations separately. Site frequency spectrum (SFS) of polymorphic sites at the *myb114* gene was plotted for the two populations separately. Linkage disequilibrium (*r*^*2*^) between paired polymorphic sites was computed using Haploview software version 4.2 (http://www.broadinstitute.org/haploview). The minimum number of recombination events was calculated with DnaSP.

Furthermore, two recently developed summary statistics, β^(2)^ and Non-central Deviation (NCD2) [20, 21, 47], were used to scan the genomes of each of the three populations for signatures of balancing selection. These methods have been shown to be more powerful and robust than traditional tests in avoiding the influence of non-equilibrium demographic histories. High β^(2)^ scores suggest an excess of SNPs at similar frequencies [20, 47], while low NCD2 scores suggest a build-up of SNPs near a specified intermediate frequency [21]. For resequencing data for Jianshan population, mapping-generated bam file was converted into formats suitable for β^(2)^ and NCD2 score calculations using vcftools [102], glactools [103] and a python script (baypass2betascan2.py [104]). For Pool-seq data from Dishuiyan and Maofengshan populations, mapping-generated pileup files were combined according to location and then converted into formats suitable for β^(2)^ and NCD2 score calculations using popololation2, poolfstat (https://cran.r-project.org/web/packages/poolfstat/index.html) and the python script baypass2betascan2.py. At the genome level, all SNPs with a MAF > 0.05 in each population were used for the β^(2)^ and NCD2 statistics. SNP frequencies were polarized and substitutions were called using *M. dodecandrum* as the outgroup. β^(2)^ scores were calculated using Betascan2 [47] with DivTime set to 2 and window size set to 1 kb. NCD2 scores were calculated using a python script (NCD_snpwise.py [104]) with window size set to 1 kb and target frequency set to 0.5. β^(2)^ and NCD2 scores for *myb114* in each of the three population were extracted according to its genomic position from its start codon to stop codon. Statistical significance in the β^(2)^ and NCD2 scores between the genome averages and *myb114* was carried out using the Wilcoxon test. The results were plotted using geom_density2d function in the R package ggplot2.

### Nucleotide diversity and polymorphism analysis of *myb114* and surrounding regions

To compare the nucleotide diversity and polymorphism of *myb114* versus surrounding regions, six single-copy segments distributed across a ~60-kb region centered on *myb114* were amplified and sequenced with specific primers (Table S12). The randomly sampled individuals of *M. normale* from Maofengshan population were partitioned according to *myb114* genotype. Fourteen individuals with RR genotype and 12 individuals with WW genotype (see the “Results” section for genotype assignment) were used for calculating nucleotide diversity and polymorphism for each of the six segments and *myb114* in DnaSP. Statistical significance on nucleotide diversity and polymorphism between *myb114* and six surrounding segments was conducted using one sample *t* test.

## Supplementary Information

**Additional file 1: Table S1.** Sampling information in this study. **Table S2.** Sequencing and mapping statistics of four pooled population samples of *M. normale. ***Table S3.** Population genetic statistics for four pooled population samples of *M. mormale *with a 5-Kb sliding window analysis. **Table S4.** Pairwise genetic differentiationacross the genome for four pooled population samples of *M. mormale* with a 5-Kb sliding window analysis. **Table S5.** SNP sites with the top 50 Fst values in the genome between the Wite and Red morphs of *M. normale* in Dishuiyan. **Table S6.** SNP sites with the top 50 Fst values in the genome between the Wite and Red morphs of *M. normale* in Maofengshan. **Table S7.** Pairwise genetic differentiationat the *myb114* gene for four pooled population samples of *M. mormale *using Sanger sequencing. **Table S8.** Genotype counts and Fisher’s exact tests for association between haplogroup and twig trichome color in Dishuiyan and Maofengshan populationsof *Melastoma normale*. **Table S9.** The frequency of two main haplotypes of the *myb114* gene in different morphs of *M. normale* in Maofengshan and Dishuiyan populations. **Table S10.** Nucleotide diversityand polymorphismof the *myb114* gene and six surrounding segments in *M. normale* in the Maofengshan population. **Table S11.** Statistical significance on nucleotide diversityand polymorphismbetween the *myb114* gene and six surrounding segments in *M. normale* in the Maofengshan population. **Table S12.** Specific primers for *Melastoma* species used in this paper. **Fig. S1.** The nucleotide diversityof four pooled population samples of *M. normale* across the genome. **Fig. S2.** The nucleotide polymorphismof four pooled population samples of *M. normale* across the genome. **Fig. S3.** Tajima’s D of four pooled population samples of *M. normale* across the genome. **Fig. S4.** Genetic differentiationbetween the same morphs sampled from Maofengshan and Dishuiyan based on a sliding window analysis of Pool-seq data. **Fig. S5.** The mapping depth of nucleotide positions 500000-525000 on scaffold27 for three species of *Melastoma*, namely, *M. candidum*, *M. normale* and *M. dodecandrum*. **Fig. S6.** Site frequency spectrum of the *myb* gene in Dishuiyanand Maofengshanpopulations of *M. normale*. **Fig. S7.** Haploview plot depicting the haplotype block structure of the *myb114* gene for Dishuiyanand Maofengshanpopulations of *M. normale*. **Fig. S8.** *β* and NCD2 score density plot for three populations of *Melastoma normale*. **Fig. S9.** Nucleotide diversity and polymorphism of *myb114* and surrounding regions in the Maofengshan population of *Melastoma normale*. **Fig. S10.** The mapping depth of nucleotide positions 500000-520000 on scaffold27 for one sample of *M. candidum* and two pooled population samples of *M. normale* from Dishuiyan. **Fig. S11.** Structural variation of the regions containing the *myb114* gene observed in *M. normale *relative to *M. candidum*.** SI text 1.** Characterization of copy number of *myb114* in six species of *Melastoma*.

## Data Availability

The datasets supporting the conclusions of this article are available in the GenBank repository [MK618466 - MK618508, MT010136 - MT010215, SRR8892966 - SRR8892971 and SRR19183013 - SRR19183030]. Genome sequence data of four *Melastoma* species are available in GenBank under accession numbers JARUQA000000000, JARUPX000000000, JARUPY000000000, and JARUPW000000000.
