# Supplementary material for: Balancing selection on an MYB transcription factor maintains the twig trichome color variation in Melastoma normale
Source: BMC Biol. 2023 May 24;21:122. doi: 10.1186/s12915-023-01611-4 (PMC10210440; doi:10.1186/s12915-023-01611-4)
Supplement: Supplementary file 1 — Additional file 1: Table S1. Sampling information in this study. Table S2. Sequencing and mapping statistics of four pooled population samples of M. normale. Table S3. Population genetic statistics for four pooled population samples of M. mormale with a 5-Kb sliding window analysis. Table S4. Pairwise genetic differentiationacross the genome for four pooled population samples of M. mormale with a 5-Kb sliding window analysis. Table S5. SNP sites with the top 50 Fst values in the genome between the Wite and Red morphs of M. normale in Dishuiyan. Table S6. SNP sites with the top 50 Fst values in the genome between the Wite and Red morphs of M. normale in Maofengshan. Table S7. Pairwise genetic differentiationat the myb114 gene for four pooled population samples of M. mormale using Sanger sequencing. Table S8. Genotype counts and Fisher’s exact tests for association between haplogroup and twig trichome color in Dishuiyan and Maofengshan populationsof Melastoma normale. Table S9. The frequency of two main haplotypes of the myb114 gene in different morphs of M. normale in Maofengshan and Dishuiyan populations. Table S10. Nucleotide diversityand polymorphismof the myb114 gene and six surrounding segments in M. normale in the Maofengshan population. Table S11. Statistical significance on nucleotide diversityand polymorphismbetween the myb114 gene and six surrounding segments in M. normale in the Maofengshan population. Table S12. Specific primers for Melastoma species used in this paper. Fig. S1. The nucleotide diversityof four pooled population samples of M. normale across the genome. Fig. S2. The nucleotide polymorphismof four pooled population samples of M. normale across the genome. Fig. S3. Tajima’s D of four pooled population samples of M. normale across the genome. Fig. S4. Genetic differentiationbetween the same morphs sampled from Maofengshan and Dishuiyan based on a sliding window analysis of Pool-seq data. Fig. S5. The mapping depth of nucleotide positions 50000 [file 12915_2023_1611_MOESM1_ESM.docx]

**Additional file 1 for**

**Balancing selection on an myb transcription factor maintains the twig trichome color variation in *Melastoma normale***

Guilian Huang^1#^, Wei Wu^1#^, Yongmei Chen^2#^, Xueke Zhi^1^, Peishan Zou^1^, Zulin Ning^3^, Qiang Fan^1^, Ying Liu^1^, Shulin Deng^3^, Kai Zeng^4^*, Renchao Zhou^1^*

**Contents**

**Table S1-S12** **……………………………………………….……………….…….2-16**

**Fig. S1-S11………………………………………………………………..……….17-27**

**SI text 1……………….…………………………………………………..……….28-30**

**Table S1 Sampling information in this study**

| **Taxon** | **Location** | **Sample size** | **GenBank accession number** |  |
| --- | --- | --- | --- | --- |
| *M. normale* | Dishuiyan Forest Park, Panyu District, Guangzhou, Guangdong | 30 (Red morph, used for Pool-seq)  30 (White morph, used for Pool-seq) | SRR8892970  SRR8892971 |  |
|  | Maofengshan Forest Park, Baiyun District, Guangzhou, Guangdong | 24 (Red morph, used for Pool-seq)  25 (White morph, used for Pool-seq) | SRR8892968  SRR8892969 |  |
|  | Dishuiyan Forest Park, Panyu District, Guangzhou, Guangdong | 50 (random sampling) | MK618496-MK618497; MK618486-MK618487 |  |
|  | Maofengshan Forest Park, Baiyun District, Guangzhou, Guangdong | 50 (random sampling) | MK618485-MK618489; MK618493; MK618496-MK618503 |  |
|  | Jianshan, Zigong, Sichuan | 20 (random sampling, 15 used for genome resequencing) | SRR19183013-SRR19183020; SRR19183023-SRR19183029 |  |
|  | Sun Yat-sen University Campus, Zhuhai, Guangdong | 1 (genome resequencing) | SRR19183030 |  |
| *M. dodecandrum* | Yucangshan, Cangnan, Zhejiang | 5 (1 used for genome resequencing) | SRR19183021; MK618466-MK618467 |  |
| *M. candidum* | Ziyun Park, Longhai, Fujian | 5 (1 used for genome resequencing) | SRR19183022; MK618484 |  |
| *M. sanguineum* | Wangzishan, Huadu District, Guangzhou, Guangdong | 5 | MK618470-MK618471 |  |
| *M. penicillatum* | Wuzhishan, Qiongzhong, Hainan | 5 | MK618477- MK618483 |  |
| *M. malabathricum* | Tianjingshan, Ruyuan, Guangdong | 5 | MK618472-MK618476 |  |
| *Osbeckia stellata* | Hulou, Chishui, Guangzhou | 1 | ON565433 |  |

**Table S2 Sequencing and mapping statistics of four pooled population samples of *M. normale***

| **Pooled population sample** | **No. of raw sequence reads** | **No. of sequence reads after quality filtering** | **Average depth** | **Percentage of mapped reads** |
| --- | --- | --- | --- | --- |
| Maofengshan_Red morph | 263,501,960 | 226,634,764 | 130.74× | 96.56% |
| Maofengshan_White morph | 266,238,904 | 231,087,574 | 136.70× | 96.11% |
| Dishuiyan_Red morph | 286,492,238 | 246,688,020 | 127.42× | 95.55% |
| Dishuiyan_White morph | 276,695,940 | 235,156,432 | 126.09× | 96.33% |

**Table S3 Population genetic statistics for four pooled population samples of *M. mormale* with a 5-Kb sliding window analysis. Mean and standard deviation (in parenthesis) was shown for each parameter.**

| Pooled population sample | **Nucleotide diversity (π)** | **Nucleotide polymorphism (θw)** | **Tajima’s D** |
| --- | --- | --- | --- |
| Maofengshan_Red morph | 0.0093 (±0.0060) | 0.0107 (±0.0064) | -0.6381 (±0.6633) |
| Maofengshan_White morph | 0.0094 (±0.0060) | 0.0107 (±0.0064) | -0.6146 (±0.6781) |
| Dishuiyan_Red morph | 0.0083 (±0.0058) | 0.0084 (±0.0056) | -0.1571 (±0.8752) |
| Dishuiyan_White morph | 0.0082 (±0.0058) | 0.0083 (±0.0056) | -0.1935 (±0.8800) |

**Table S4 Pairwise genetic differentiation (Fst) across the genome for four pooled population samples of *M. mormale* with a 5-Kb sliding window analysis**

| **Pooled population sample** | Maofengshan_White morph | Maofengshan_Red morph | Dishuiyan_White morph |
| --- | --- | --- | --- |
| Maofengshan_Red morph | 0.016 (±0.011) | - | - |
| Dishuiyan_White morph | 0.063 (±0.038) | 0.063 (±0.037) | - |
| Dishuiyan_Red morph | 0.064 (±0.038) | 0.063 (±0.038) | 0.017 (±0.008) |

**Table S5 SNP sites with the top 50 Fst values in the genome between the Wite and Red morphs of *M. normale* in Dishuiyan**

| **Site No.** | **Scaffold** | **Location** | **Depth** | **Fst** |
| --- | --- | --- | --- | --- |
| 1 | Scaffold27 | 503308 | 88 | 0.916 |
| 2 | Scaffold27 | 504872 | 83 | 0.816 |
| 3 | Scaffold27 | 503603 | 73 | 0.808 |
| 4 | Scaffold27 | 503450 | 89 | 0.804 |
| 5 | Scaffold27 | 504790 | 91 | 0.801 |
| 6 | Scaffold27 | 505924 | 62 | 0.800 |
| 7 | Scaffold27 | 504918 | 81 | 0.777 |
| 8 | Scaffold27 | 508157 | 103 | 0.768 |
| 9 | Scaffold27 | 503685 | 74 | 0.764 |
| 10 | Scaffold27 | 505173 | 88 | 0.757 |
| 11 | Scaffold27 | 505087 | 90 | 0.749 |
| 12 | Scaffold27 | 504579 | 63 | 0.735 |
| 13 | Scaffold27 | 504662 | 72 | 0.735 |
| 14 | Scaffold27 | 506366 | 74 | 0.728 |
| 15 | Scaffold27 | 505328 | 71 | 0.726 |
| 16 | Scaffold27 | 505280 | 71 | 0.719 |
| 17 | Scaffold27 | 505281 | 71 | 0.719 |
| 18 | Scaffold27 | 508182 | 100 | 0.718 |
| 19 | Scaffold27 | 505304 | 74 | 0.717 |
| 20 | Scaffold27 | 506365 | 75 | 0.712 |
| 21 | Scaffold27 | 505127 | 96 | 0.707 |
| 22 | Scaffold27 | 505091 | 88 | 0.703 |
| 23 | Scaffold27 | 505058 | 96 | 0.697 |
| 24 | Scaffold27 | 510578 | 102 | 0.691 |
| 25 | Scaffold27 | 506116 | 80 | 0.687 |
| 26 | Scaffold27 | 508131 | 105 | 0.686 |
| 27 | Scaffold27 | 503681 | 70 | 0.682 |
| 28 | Scaffold27 | 508432 | 90 | 0.681 |
| 29 | Scaffold27 | 506146 | 73 | 0.677 |
| 30 | Scaffold27 | 508778 | 92 | 0.673 |
| 31 | Scaffold27 | 506407 | 75 | 0.671 |
| 32 | Scaffold27 | 506043 | 80 | 0.670 |
| 33 | Scaffold27 | 506409 | 76 | 0.670 |
| 34 | Scaffold27 | 506410 | 75 | 0.668 |
| 35 | Scaffold27 | 506345 | 73 | 0.667 |
| 36 | Scaffold27 | 504343 | 78 | 0.667 |
| 37 | Scaffold27 | 504061 | 94 | 0.664 |
| 38 | Scaffold27 | 506065 | 75 | 0.661 |
| 39 | Scaffold27 | 504711 | 73 | 0.659 |
| 40 | Scaffold27 | 508212 | 97 | 0.657 |
| 41 | Scaffold27 | 511542 | 64 | 0.656 |
| 42 | Scaffold27 | 504825 | 95 | 0.655 |
| 43 | Scaffold27 | 505209 | 85 | 0.655 |
| 44 | Scaffold27 | 506207 | 72 | 0.653 |
| 45 | Scaffold27 | 504832 | 93 | 0.653 |
| 46 | Scaffold27 | 506067 | 75 | 0.649 |
| 47 | Scaffold27 | 506017 | 81 | 0.649 |
| 48 | Scaffold27 | 506162 | 72 | 0.648 |
| 49 | Scaffold27 | 506058 | 80 | 0.646 |
| 50 | Scaffold27 | 506448 | 84 | 0.640 |

**Table S6 SNP sites with the top 50 Fst values in the genome between the Wite and Red morphs of *M. normale* in Maofengshan**

| **Site No.** | **Scaffold** | **Location** | **Depth** | **Fst** |
| --- | --- | --- | --- | --- |
| 1 | Scaffold27 | 504220 | 71 | 0.668 |
| 2 | Scaffold27 | 503308 | 88 | 0.600 |
| 3 | Scaffold27 | 509515 | 81 | 0.573 |
| 4 | Scaffold27 | 503681 | 70 | 0.562 |
| 5 | Scaffold27 | 509731 | 84 | 0.541 |
| 6 | Scaffold27 | 504832 | 93 | 0.537 |
| 7 | Scaffold27 | 504204 | 71 | 0.531 |
| 8 | Scaffold27 | 504061 | 94 | 0.526 |
| 9 | Scaffold27 | 504825 | 95 | 0.518 |
| 10 | Scaffold27 | 508778 | 92 | 0.515 |
| 11 | Scaffold27 | 508272 | 103 | 0.512 |
| 12 | Scaffold27 | 504807 | 96 | 0.511 |
| 13 | Scaffold27 | 503450 | 89 | 0.507 |
| 14 | Scaffold27 | 503603 | 73 | 0.505 |
| 15 | Scaffold27 | 506146 | 73 | 0.492 |
| 16 | Scaffold27 | 507962 | 102 | 0.491 |
| 17 | Scaffold27 | 508182 | 100 | 0.485 |
| 18 | Scaffold27 | 505980 | 61 | 0.482 |
| 19 | Scaffold27 | 508269 | 99 | 0.481 |
| 20 | Scaffold27 | 505924 | 62 | 0.477 |
| 21 | Scaffold27 | 505087 | 90 | 0.475 |
| 22 | Scaffold27 | 504872 | 83 | 0.470 |
| 23 | Scaffold27 | 508432 | 90 | 0.469 |
| 24 | Scaffold27 | 505091 | 88 | 0.464 |
| 25 | Scaffold27 | 508131 | 105 | 0.462 |
| 26 | Scaffold27 | 505058 | 96 | 0.457 |
| 27 | Scaffold0 | 9337996 | 74 | 0.452 |
| 28 | Scaffold27 | 505280 | 71 | 0.449 |
| 29 | Scaffold27 | 505281 | 71 | 0.449 |
| 30 | Scaffold27 | 504711 | 73 | 0.432 |
| 31 | Scaffold27 | 508157 | 103 | 0.426 |
| 32 | Scaffold27 | 506407 | 75 | 0.421 |
| 33 | Scaffold27 | 506017 | 81 | 0.416 |
| 34 | Scaffold27 | 506448 | 84 | 0.412 |
| 35 | Scaffold27 | 508949 | 72 | 0.410 |
| 36 | Scaffold27 | 506410 | 75 | 0.409 |
| 37 | Scaffold27 | 504790 | 91 | 0.408 |
| 38 | Scaffold27 | 505304 | 74 | 0.398 |
| 39 | Scaffold27 | 504343 | 78 | 0.395 |
| 40 | Scaffold27 | 508212 | 97 | 0.395 |
| 41 | Scaffold27 | 505127 | 96 | 0.394 |
| 42 | Scaffold27 | 505328 | 71 | 0.394 |
| 43 | Scaffold35 | 1612634 | 63 | 0.377 |
| 44 | Scaffold27 | 504405 | 87 | 0.371 |
| 45 | Scaffold27 | 506365 | 75 | 0.370 |
| 46 | Scaffold35 | 1612628 | 63 | 0.369 |
| 47 | Scaffold27 | 504918 | 81 | 0.368 |
| 48 | Scaffold4 | 3214246 | 64 | 0.368 |
| 49 | Scaffold27 | 509375 | 94 | 0.367 |
| 50 | Scaffold27 | 506366 | 74 | 0.367 |

**Table S7 Pairwise genetic differentiation (Fst) at the *myb114* gene for four pooled population samples of *M. mormale* using Sanger sequencing**

| **Pooled population sample** | **Maofengshan**  **(White morph)** | **Maofengshan**  **(Red morph)** | **Dishuiyan**  **(White morph)** |
| --- | --- | --- | --- |
| Maofengshan  (Red morph) | 0.557 | - | - |
| Dishuiyan  (White morph) | 0.0526 | 0.736 | - |
| Dishuiyan  (Red morph) | 0.719 | 0.0521 | 0.872 |

**Table S8 Genotype counts and Fisher’s exact tests for association between haplogroup and twig trichome color in Dishuiyan and Maofengshan populations (Pool-seq) of *Melastoma normale*. See the main text for genotype definition.**

| **Population** | **Genotype** | | **Twig trichome color-White** | **Twig trichome color-Red** | **P value** |
| --- | --- | --- | --- | --- | --- |
| Dishuiyan | | WW | 23 | 0 | 1.541e-14 |
|  |  | WR | 4 | 2 |  |
|  |  | RR | 0 | 24 |  |
| Maofengshan | | WW | 15 | 0 | 1.118e-06 |
|  |  | WR | 2 | 6 |  |
|  |  | RR | 3 | 13 |  |

**Table S9 The frequency of two main haplotypes of** **the *myb114* gene in different morphs of *M. normale* in Maofengshan and Dishuiyan populations**

| **Pooled population sample** | **H1** | **H6** |
| --- | --- | --- |
| Maofengshan_White morph | 0.200 | 0.625 |
| Maofengshan_Red morph | 0.632 | 0 |
| Dishuiyan_White morph | 0.074 | 0.926 |
| Dishuiyan_Red morph | 0.923 | 0.038 |

**Table S10 Nucleotide diversity (π) and polymorphism (θw) of the *myb114* gene and six surrounding segments in *M. normale* in the Maofengshan population. Nucleotide diversity and polymorphism were calculated for 14 individuals with RR genotype and 12 with WW genotype, respectively.**

| **Segment ID (Segment length)** | ***myb114* genotype** | | **(π)** | **(θw)** |
| --- | --- | --- | --- | --- |
| S1 (811 bp) | | RR  WW | 0.00811  0.00358 | 0.00951  0.00759 |
| S2 (860 bp) | RR  WW | | 0.00334  0.00307 | 0.00269  0.00436 |
| S3 (831 bp) | RR  WW | | 0.00542  0.00483 | 0.00433  0.00419 |
| S4 (870 bp) | RR  WW | | 0.00755  0.00601 | 0.00620  0.00892 |
| S5 (844 bp) | RR  WW | | 0.00785  0.00377 | 0.00731  0.00444 |
| S6 (854 bp) | RR  WW | | 0.00639  0.00863 | 0.00512  0.00784 |
| *myb114* (1041 bp) | RR  WW | | 0.000470  0.00229 | 0.00123  0.00300 |

**Table S11 Statistical significance** **on nucleotide diversity (π) and polymorphism (θw) between the *myb114* gene and six surrounding segments in *M. normale* in the Maofengshan population. Nucleotide diversity and polymorphism were calculated for 14 individuals with RR genotype and 12 with WW genotype, respectively. One sample t test was used for this statistic.**

| **Genotype** | **Diversity/polymorphism** | **t** | **P-value** |
| --- | --- | --- | --- |
| RR | π | 8.02 | 0.00049 |
|  | θw | 4.75 | 0.0051 |
| WW | π | 3.18 | 0.025 |
|  | θw | 3.72 | 0.014 |

**Table S12 Specific primers for *Melastoma* species used in this paper**

| **Primer** | **Sequence（**5'-3'**）** | **Target region** | **Purpose** |
| --- | --- | --- | --- |
| *myb*-F | GTTCGAAGGGAGTCGACAAG | *myb114_1* | P^1^ & S^2^ |
| *myb*-R | ACGACGTTTAAGCCTTCGTG | *myb114_1* | P & S |
| Myb_1F_new | CAAAACACGAACCACAACGAAGA | *myb114_1* | P & S |
| Myb_1R_new | CAAGAACTGCCAAAGATCGATG | *myb114_1* | P & S |
| *myb*-40-1F | TAATCATGTCATGCGACGG | *myb114_1* | S |
| *myb*-40-2R | AGCGACGTTGGGTTCTGGTA | *myb114_1* | S |
| *myb*-3R | GTGAGCATGTGTGTCGGTTT | *myb114_1* | S |
| *myb*-4F | AAAAACAGACAATCCTCACACG | *myb114_1* | S |
| *myb*-5R | AGGAAGAGCTGCCGGATG | *myb114_1* | S |
| *myb*-7F | GGGGACATCGAACCCTTT | *myb114_1* | S |
| F1 | CGAGCCTAAACTGGCATGAT | 7.3 Kb region | P & S |
| R1 | GTGGTCATGCCTTGTTGATG | 7.3 Kb region | P & S |
| F2 | TGCGACGAAAAGAGAACAAA | 7.3 Kb region | P & S |
| R2 | TGATGATATTTCCCGTCATCTG | 7.3 Kb region | P & S |
| F3 | CAGATATCAGATGACGGGAAA | 7.3 Kb region | P & S |
| R3 | AAAGAGGCAAAGACGGGATT | 7.3 Kb region | P & S |
| F4 | CTGGACATGGCTTTCTTCCT | 7.3 Kb region | P & S |
| R4 | TTGGTCTTCCAGGCCTTTAC | 7.3 Kb region | P & S |
| F5 | GGTTCAGGAGACAACATCTTACG | 7.3 Kb region | P & S |
| R5 | TCTCTTAAAAATACAACCAAACCTG | 7.3 Kb region | P & S |
| F6 | AACTCTGCTCCGTCTACAAAATG | 7.3 Kb region | P & S |
| R6 | CGACAACGTCGGTTGTATGA | 7.3 Kb region | P & S |
| F7 | GCGACTTTCAACGAGGGAAG | 7.3 Kb region | P & S |
| R7 | ACCTTCCAGCAGATGATCCG | 7.3 Kb region | P & S |
| Mc_myb12_F | GCAAAACACGAACCACAACG | *myb114_1* & *myb114_2* | P & S |
| Mc_myb2_R | TTGCTCGGCCACTGAGAT | *myb114_2* | P & S |
| Mc_myb1_R | TTGCTCGGCCACTGAGCC | *myb114_1* | P & S |
| Mc_myb1_F | TCACTAAGAGCTTGGCTTGG | *myb114_1* | P & S |
| Mc_myb2_F | CTTCACTAAGAGCTTGGCTTAT | *myb114_2* | P & S |
| Mc_myb12_R | GGAGTCGACAAGAGGATGGT | *myb114_1* & *myb114_2* | P & S |
| S1_F | ACACTTGTGAAGCTGTCGGA | S1 segment | P & S |
| S1_R | GGGGACGATTCTTTTGGGGT | S1 segment | P & S |
| S1_Mid | TTGCCATCCGTGGCCATGA | S1 segment | S |
| S2_F | CCCAACCCCAAAACTCATTA | S2 segment | P & S |
| S2_R | TGACAGACATGGTGCAGGTT | S2 segment | P & S |
| S3_F | AGAAGCGAGCTGGGACAATA | S3 segment | P & S |
| S3_R | CCAGGTTGGTGAGGATGAGT | S3 segment | P & S |
| S3_Mid_F | GTTTGGGAAATCGCATGAAC | S3 segment | S |
| S3_Mid_F | CTAATTAGCCTTTCATTGGCTG | S3 segment | S |
| S4_F | CCTCCCCCATCTTATCGTCT | S4 segment | P & S |
| S4_R | TGGGCCTTTGTAACCATTTC | S4 segment | P & S |
| S4_220F | AATAACACGACGAGATTACC | S4 segment | S |
| S4_660F | CATACATTTAACTCGTTAGGAAAC | S4 segment | S |
| S4_660R | CTCAAACAATACTGACACGA | S4 segment | S |
| S4_750R | ATTATAATTCATCGATAATCATAAG | S4 segment | S |
| S4_810R | GATGTCTTACCTAATTTGATGTG | S4 segment | S |
| S5_F | AAACTCCCTTCCTCGTCGAT | S5 segment | P & S |
| S5_R | AGATACTCCCCGCTTGTGG | S5 segment | P & S |
| S6-F | CAAAGCCCTTCAAAAGCAAG | S6 segment | P & S |
| S6_R | CAAGTGCTGGGAAGTCATCA | S6 segment | P & S |
| S6_Mid | CTCGACCTTTCCTCGTCAAA | S6 segment | S |

Note: ^1^P: Used for PCR amplification; ^2^S: Used for Sequencing.


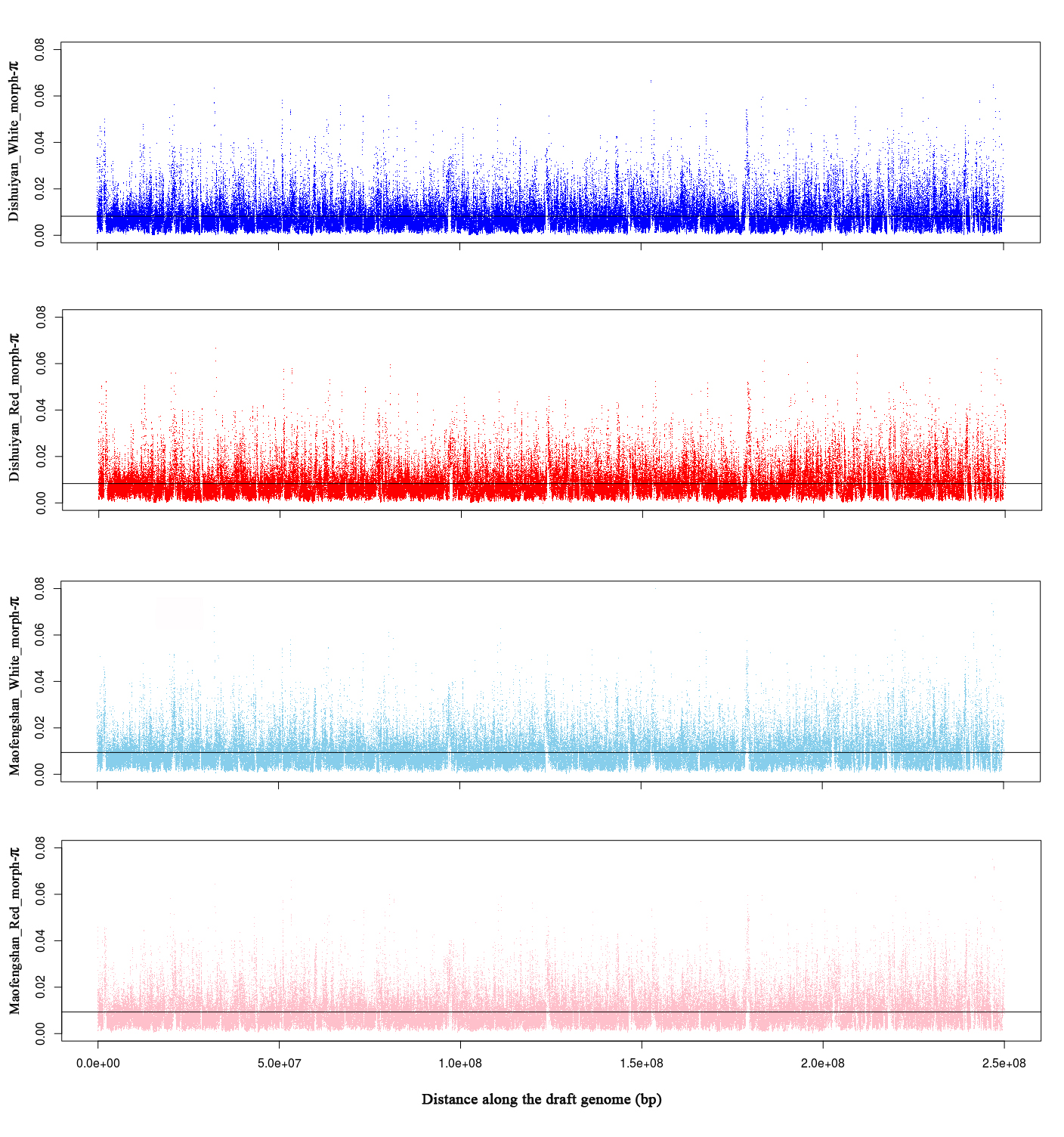


**Fig. S1** The nucleotide diversity (π) of four pooled population samples of *M. normale* across the genome. The x-axis corresponds to the reference genome, and the y-axis represents the π value. The average π value of each sample was marked by a black line. A 5-Kb sliding window analysis was performed with a step size of 1 Kb. Each dot represents the π-value of a 5-Kb window.


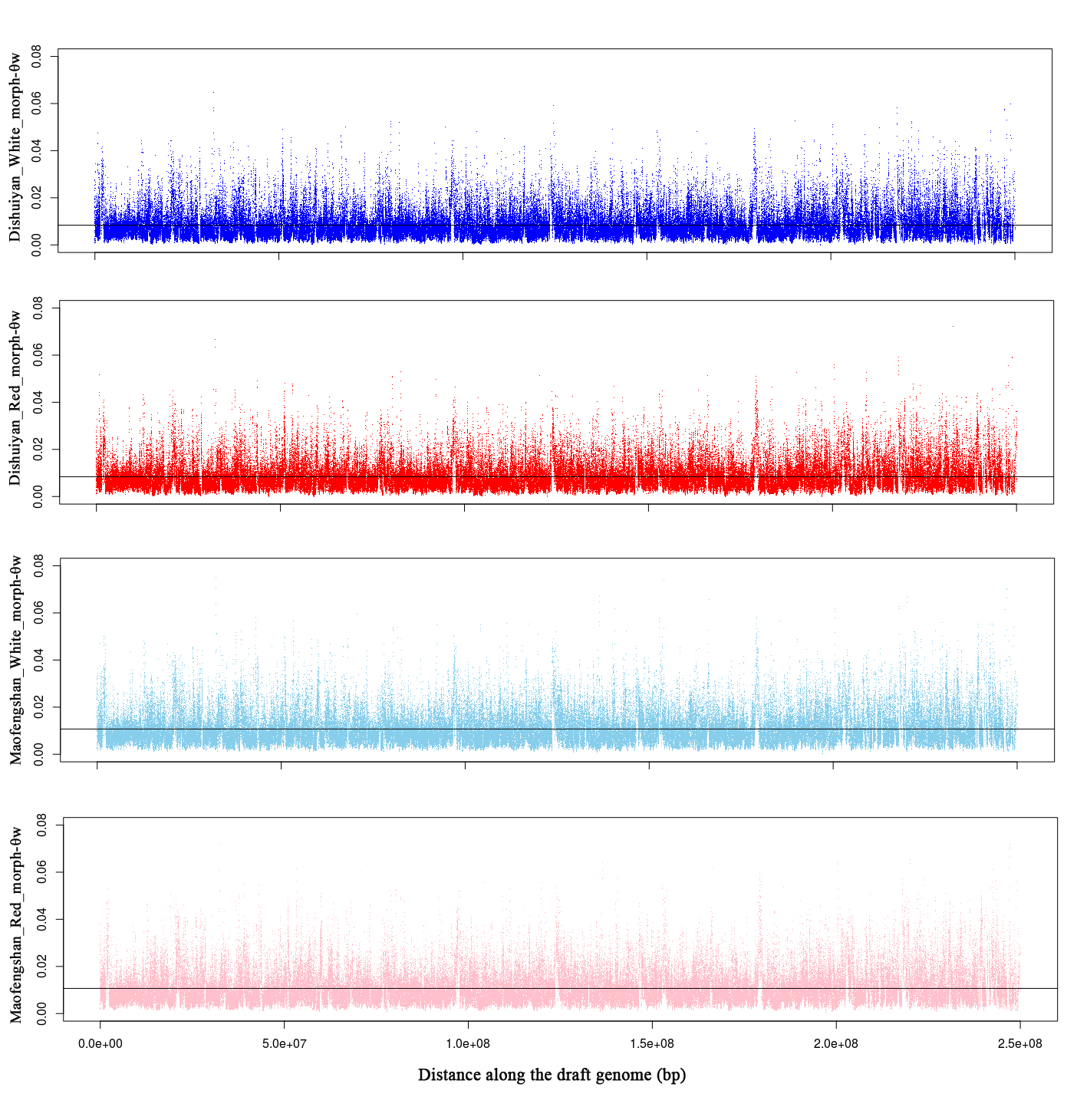


**Fig. S2** The nucleotide polymorphism (θw) of four pooled population samples of *M. normale* across the genome. The x-axis corresponds to the reference genome, and the y-axis represents the θw value. The average θw value of each sample was marked by a black line. A 5-Kb sliding window analysis was performed with a step size of 1 Kb. Each dot represents the θw value of a 5-Kb window.


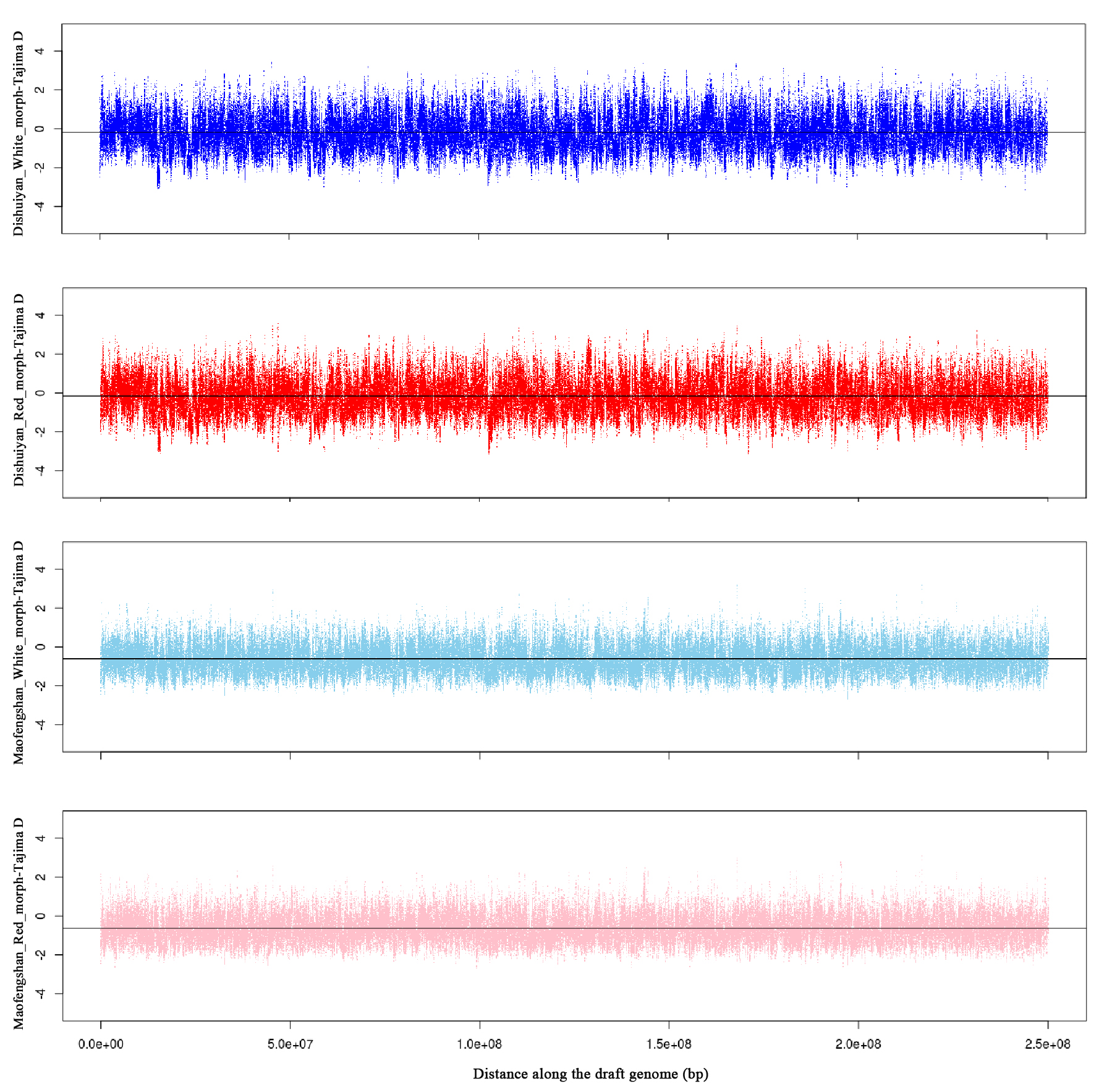


**Fig. S3** Tajima’s D of four pooled population samples of *M. normale* across the genome. The x-axis corresponds to the reference genome, and the y-axis represents the Tajima’s D value. The average Tajima’s D value of each sample was marked by a black line. A 5-Kb sliding window analysis was performed with a step size of 1 Kb. Each dot represents the Tajima’s D value of a 5-Kb window.


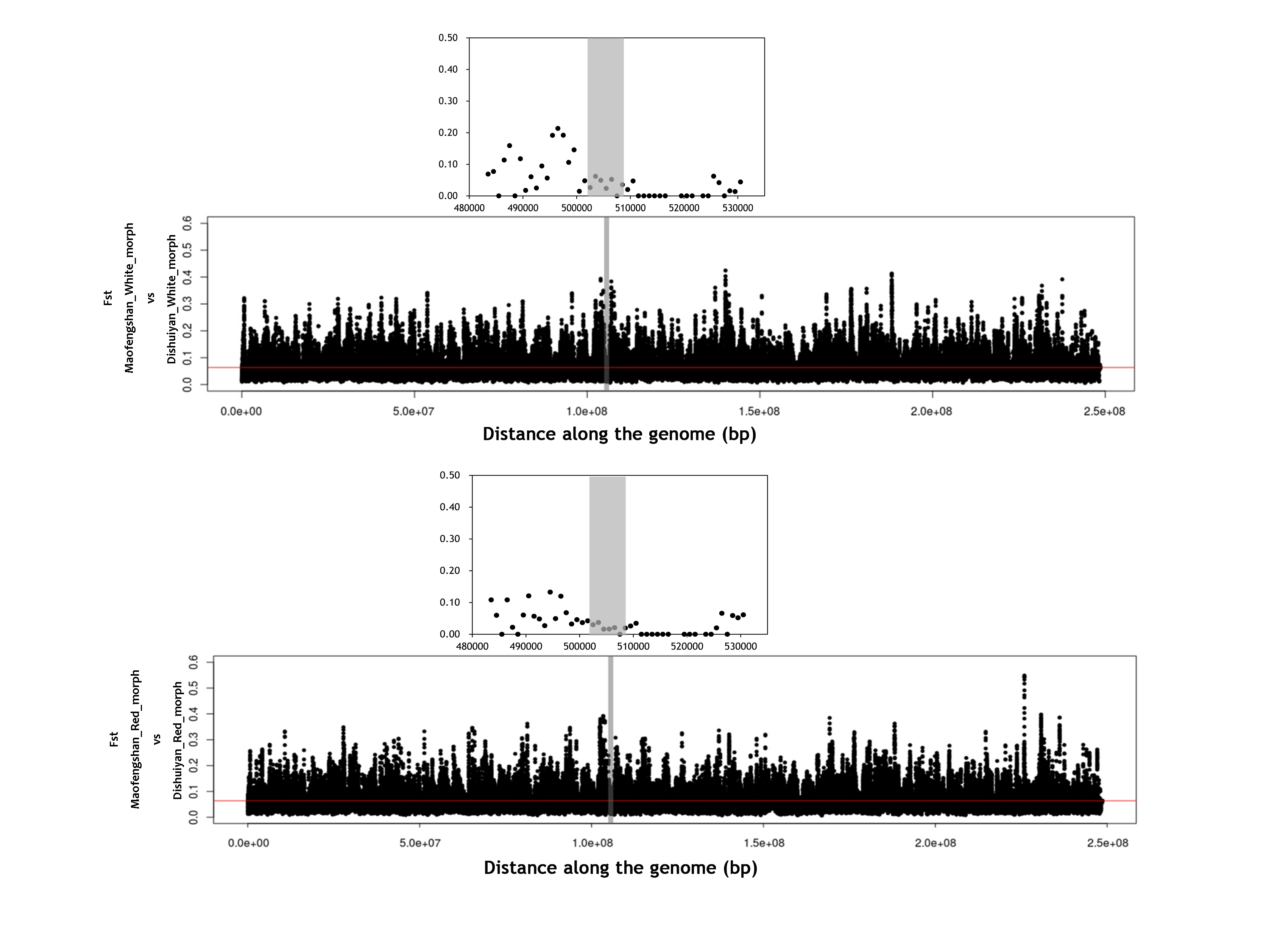


**Fig. S4** Genetic differentiation (Fst) between the same morphs sampled from Maofengshan and Dishuiyan based on a sliding window analysis of Pool-seq data. Each dot represents the Fst of a 5 Kb window. The average Fst across the genome was marked by a red line. The upper and lower panels show genetic differentiation (Fst) between the Red morphs from the two locations, and that between the White morphs from the two locations, respectively. To show the details of genetic differentiation at the 6-Kb region, Fst was also calculated with a 1 Kb sliding window and presented in a small chart for each panel. The 6 Kb region, which was highly differentiated between the two morphs, was shown in gray box.


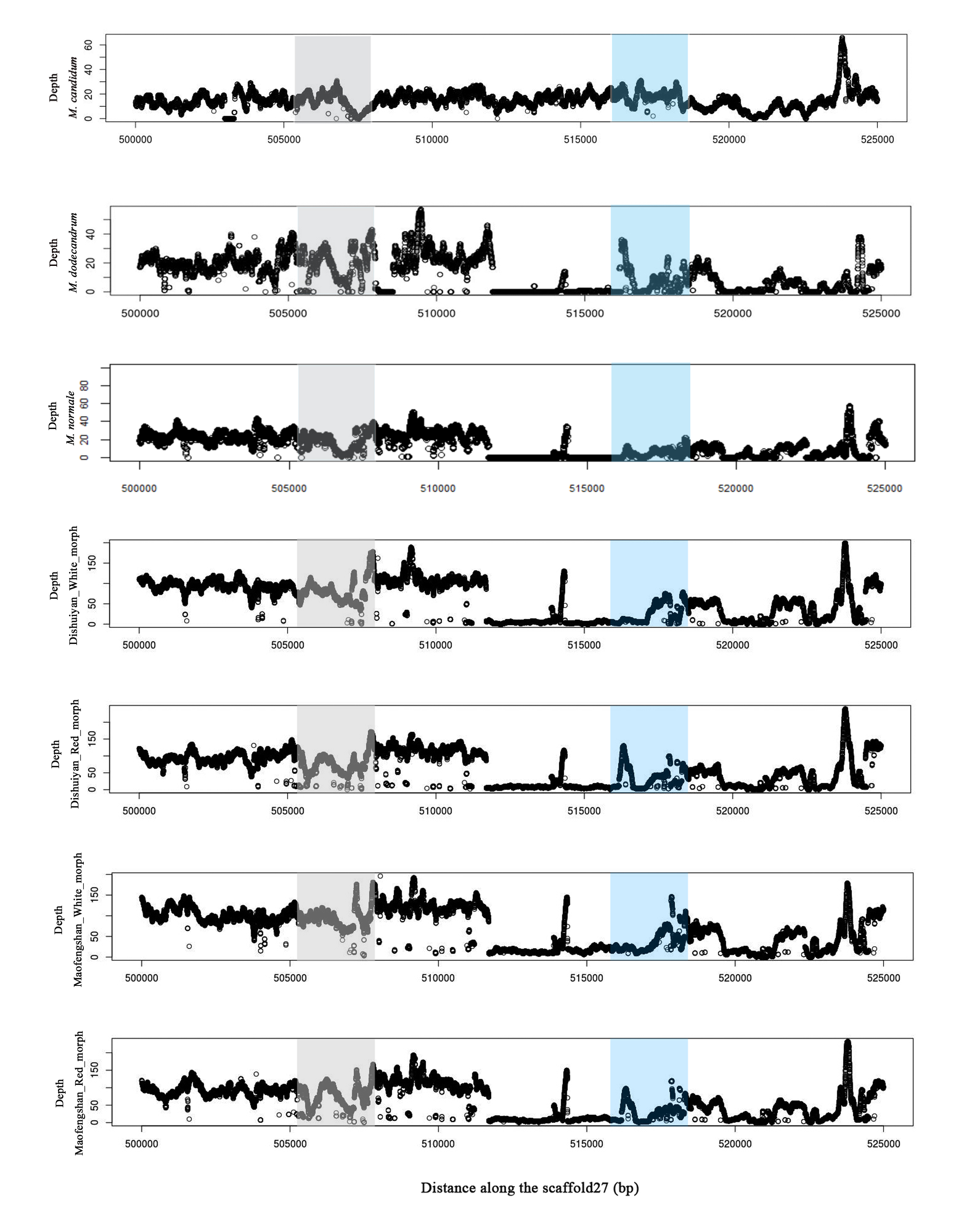


**Fig. S5** The mapping depth of nucleotide positions 500000-525000 on scaffold27 for three species of *Melastoma*, namely, *M. candidum*, *M. normale* and *M. dodecandrum*. Each black circle represents mapping depth of a site. The regions with gray and blue backgrounds represents copy1 and copy2 regions, respectively.


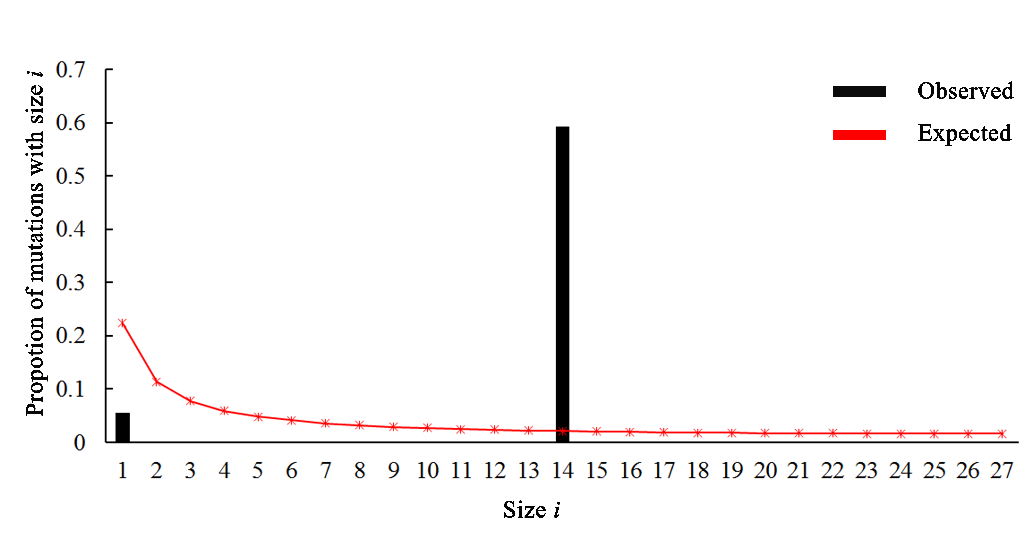

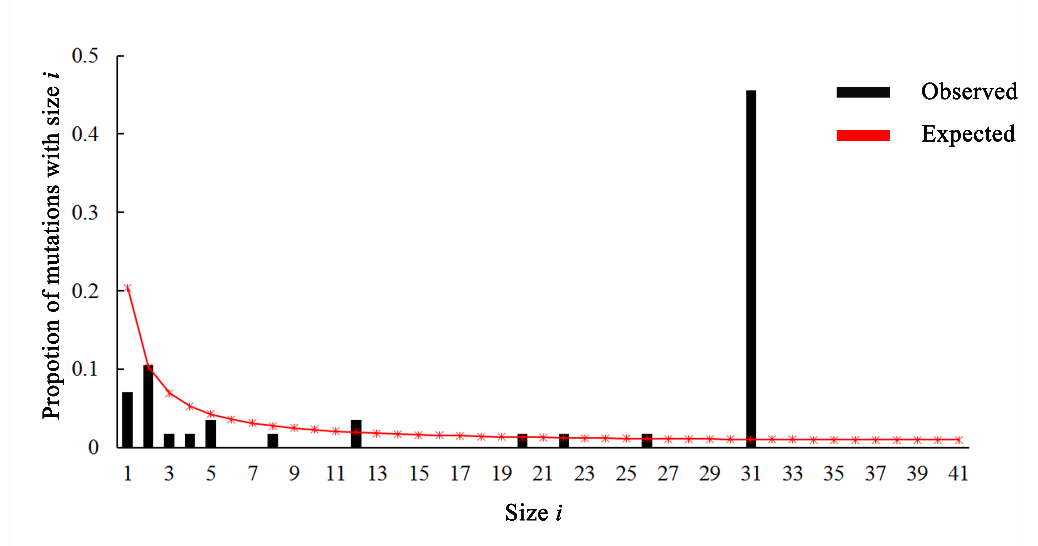


**Fig. S6** Site frequency spectrum of the *myb* gene in Dishuiyan (upper panel) and Maofengshan (lower panel) populations of *M. normale*. A mutation of size i means that it occurs i times in a population sample. The red line represents the expected frequency spectrum in neutral equilibrium, and the black bars denote the observed values.


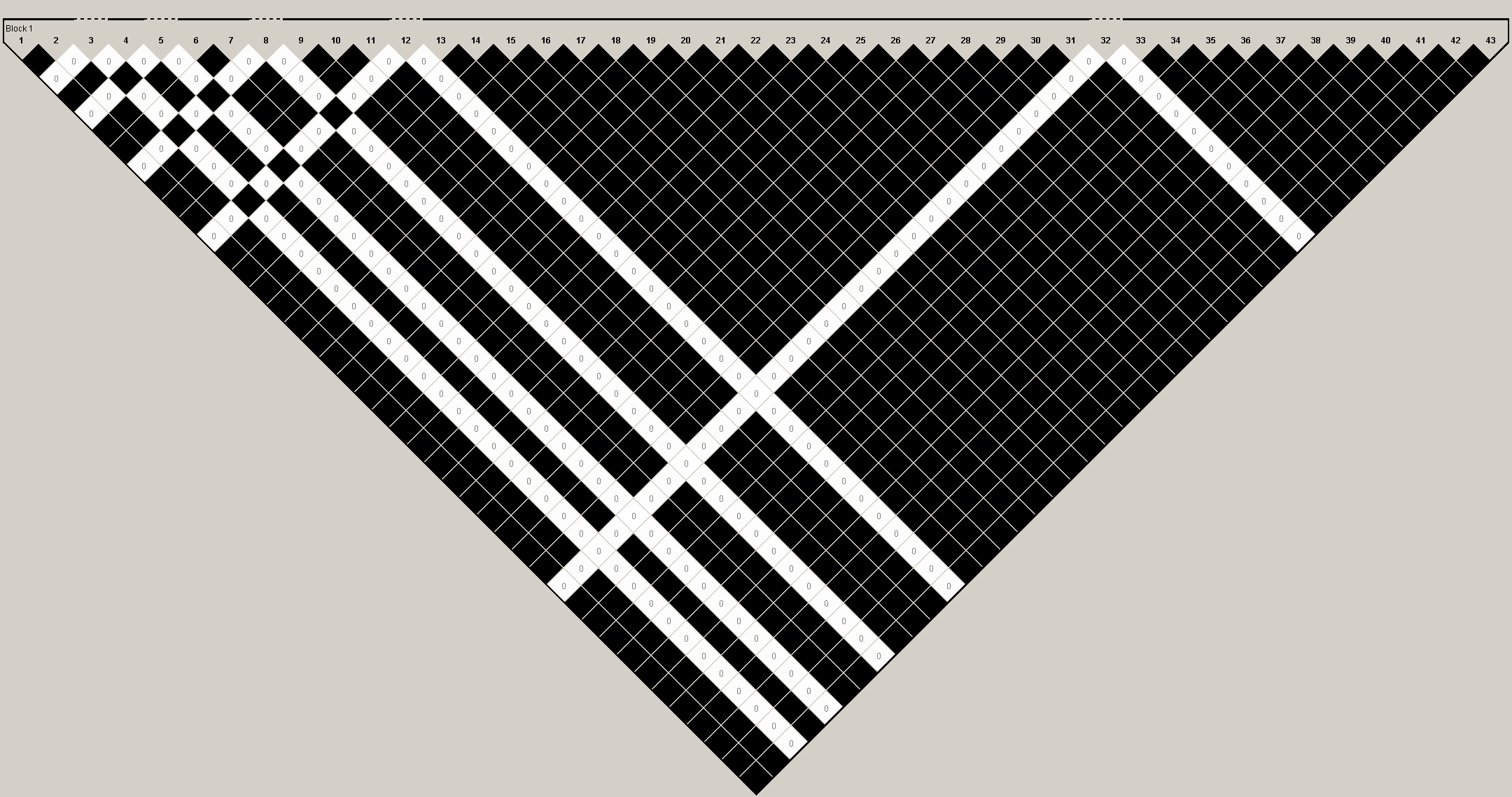


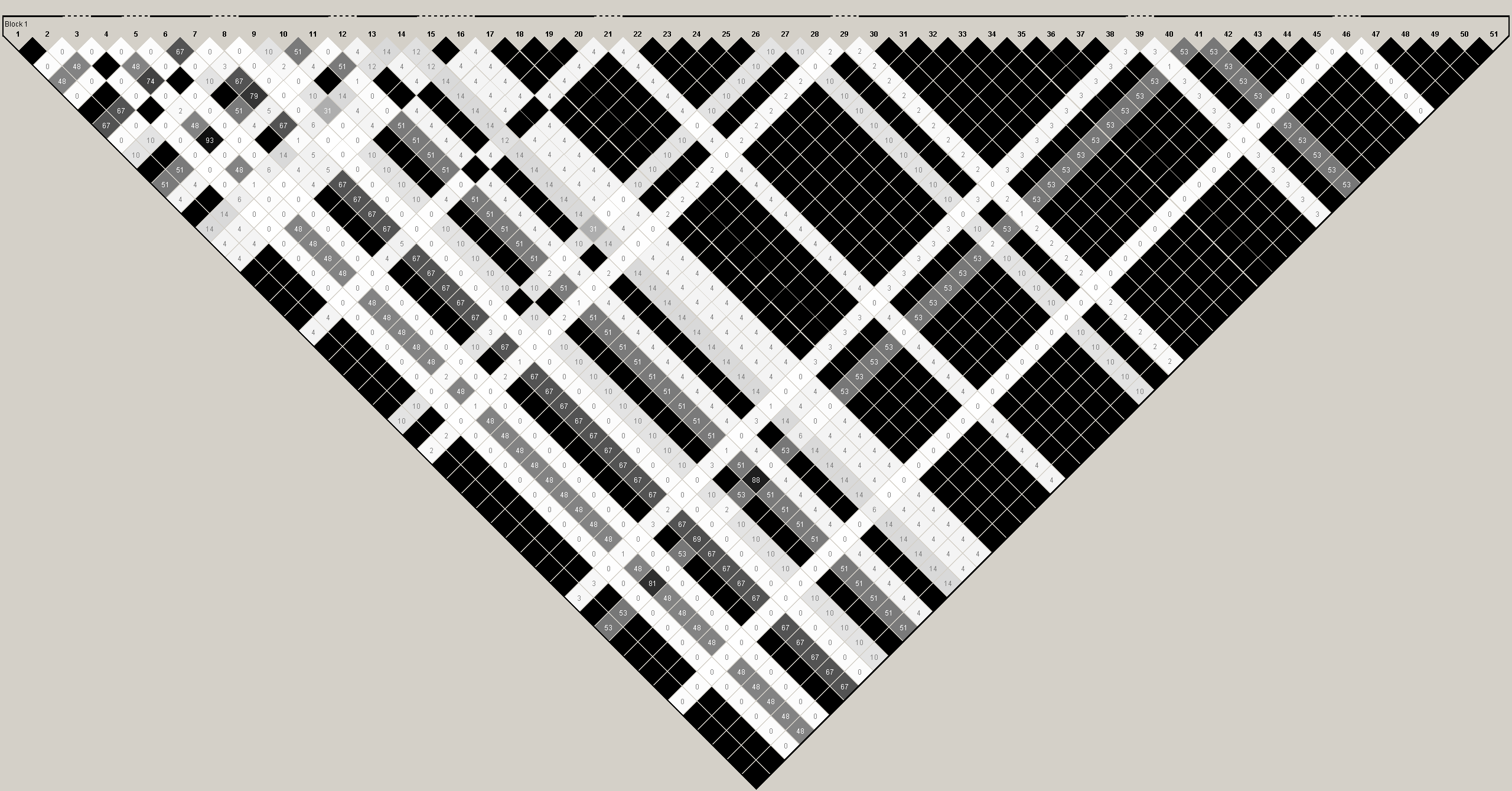


**Fig. S7** Haploview plot depicting the haplotype block structure of the *myb114* gene for Dishuiyan (upper) and Maofengshan (lower) populations of *M. normale*. Regions of LD were defined as pairwise values of the correlation coefficient (r^2^) between two segregating sites. r^2^ values from high to low were showed as black to white.

**

**

**Fig. S8** *β*^(2)^ and NCD2 score density plot for three populations of *Melastoma normale*. Higher *β*^(2)^ scores and lower NCD2 scores indicate stronger signatures of long-term balancing selection. On average, *β*^(2)^ scores are higher and NCD2 scores are lower for SNPs of *myb114* (blue) relative to those of the whole genome (red).


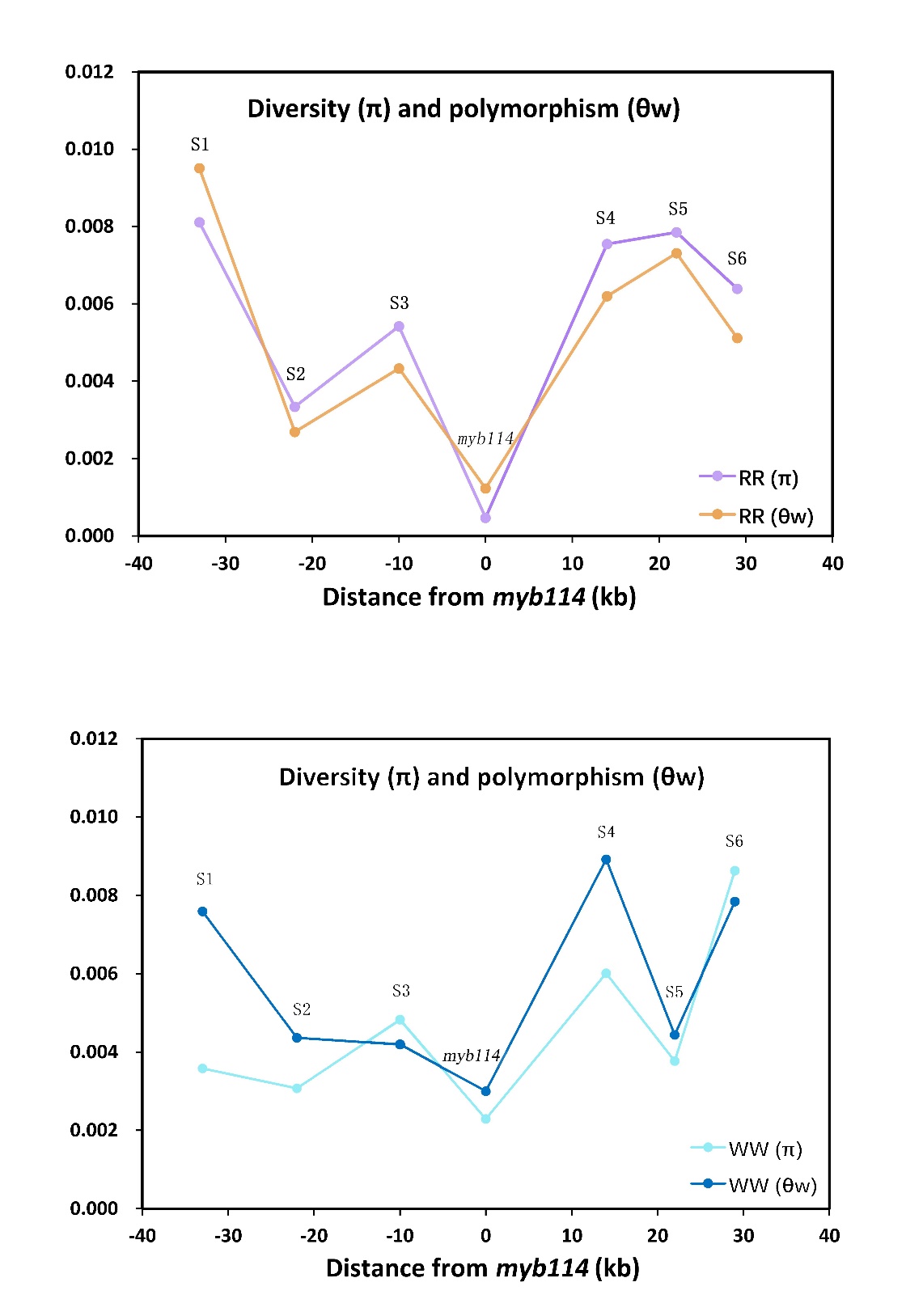


**Fig. S9** Nucleotide diversity and polymorphism of *myb114* and surrounding regions in the Maofengshan population of *Melastoma normale*. Six single-copy segments (S1-S6) distributed across a ~60 Kb region centered on *myb114* were used for comparison. The samples from the Maofengshan population are partitioned according to *myb114* genotype (RR and WW).


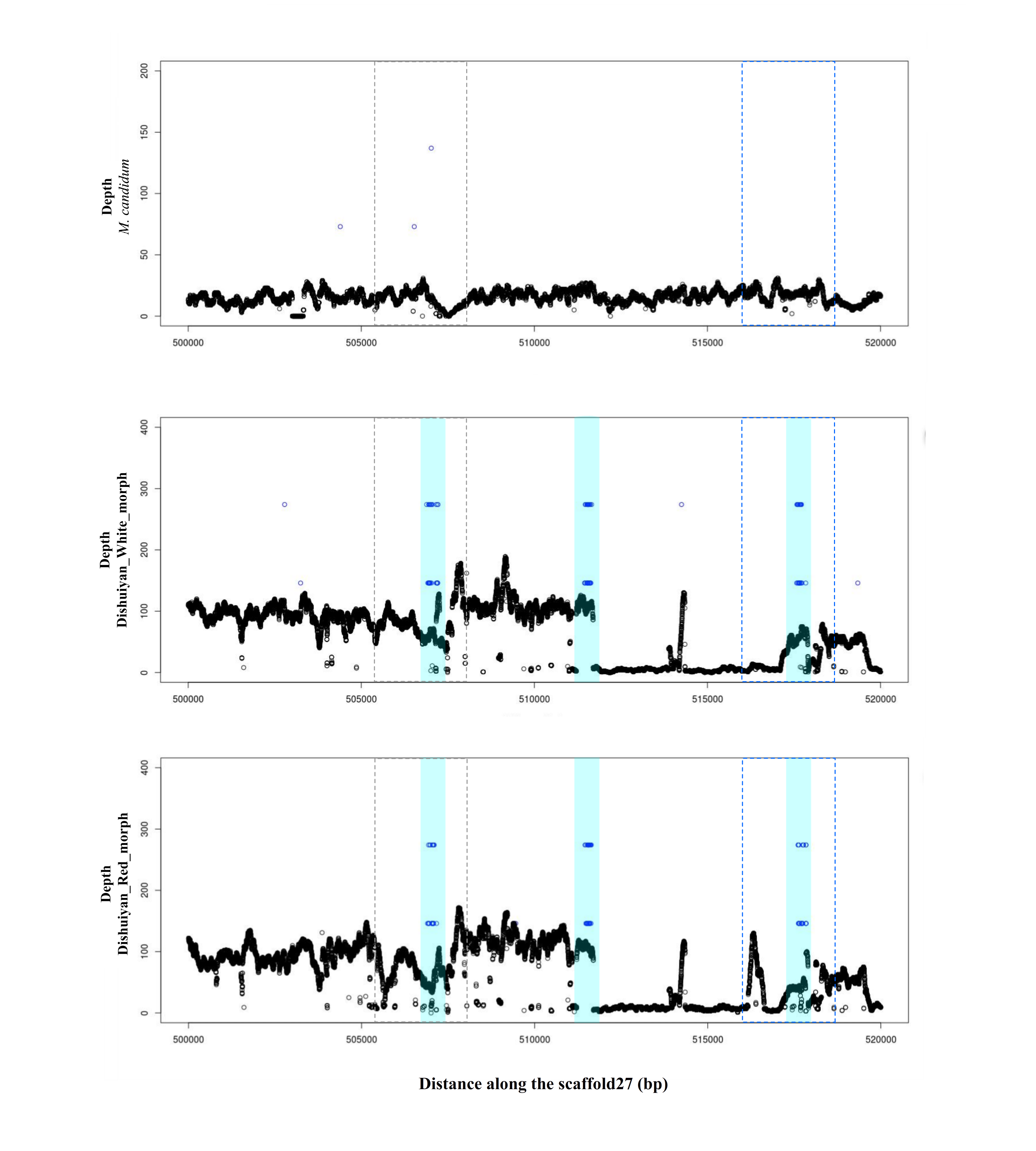


**Fig. S10** The mapping depth of nucleotide positions 500000-520000 on scaffold27 for one sample of *M. candidum* and two pooled population samples of *M. normale* from Dishuiyan. The black circle represents the mapping depth of a site and the blue circle represents the number of read pairs with only one read sucessfully mapped to the reference genome. The three regions where there are a large number of read pairs with only one read sucessfully mapped are highlighted in blue. The two box with black and blue dotted lines represent copy1 and copy2 regions, respectively.


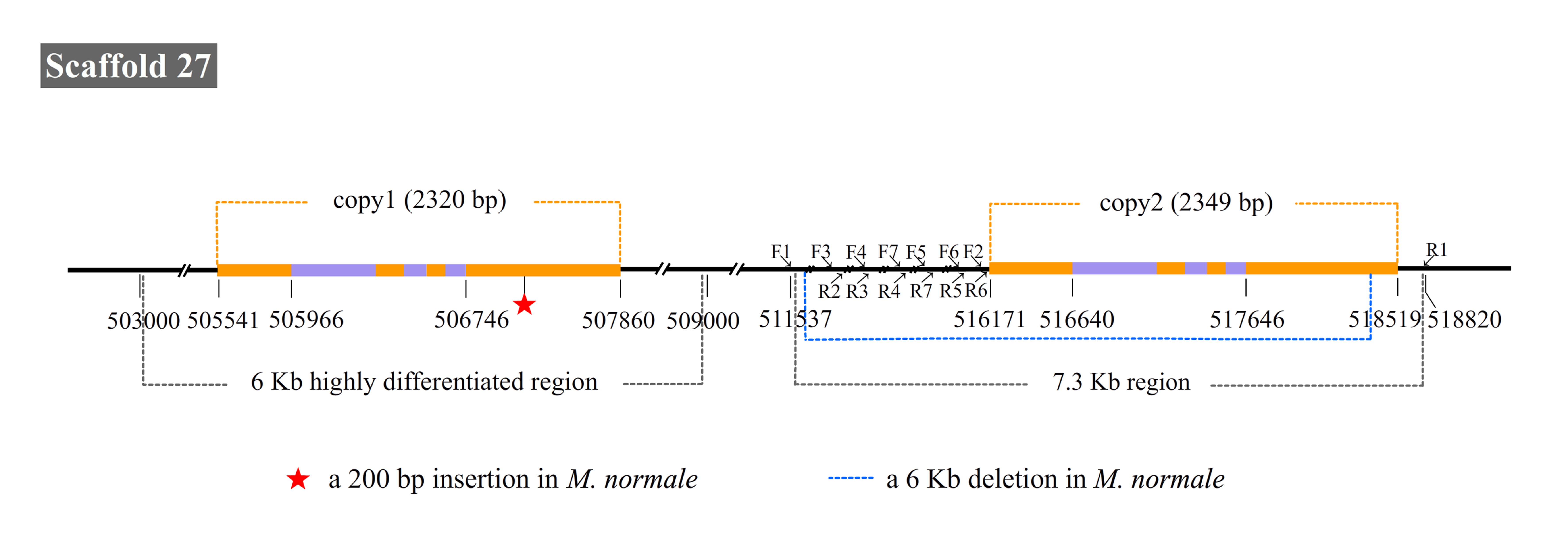


**Fig. S11** Structural variation of the regions containing the *myb114* gene observed in *M. normale* relative to *M. candidum*. The duplicate regions (copy 1 and copy 2 in yellow) are 2.3 Kb long and 10 Kb away from each other. The 6-Kb highly differentiated region in *M. normale* corresponds to the left box with black dotted line. The purple boxes in the duplicate regions represent exons of the *myb114* genes. The black arrows show the locations of specific primers in the 7.3 Kb region used for chracterization of a structural variation between *M. normale* and *M. candidum*. Structural variation observed in *M. normale* relative to *M. candidum* are shown in a red star and a box with blue dotted line.

**SI Text 1.** Characterization of copy number of *myb114* in six species of *Melastoma*.

Using PCR primers anchoring the flanking regions of *myb114-1* (Table S12), this region was amplified and sequenced for five individuals of *M. candidum* and all 109 individuals of *M. normale* used for Pool-seq. Due to high sequence identity between *myb114-1* and *myb114-2*, they were both amplified in the five individuals of *M. candidum*, with 26 double peaks in the sequence chromatograms of each individual corresponding to the sites differing between the two copies. In contrast, 67 of 109 individuals of *M. normale* were of homozygous genotype (no heterozygous sites) in this region. This suggests that *myb114* of *M. normale* might have only one copy or that there exist two highly divergent copies but only one copy was amplified here. To determine whether there is the other copy in *M. normale* or not, read depth of the 25 Kb segment containing the two copies in *M. candidum* was checked for the four Pool-seq samples of *M. normale* and an individual of *M. candidum*, and read pairs with only one read successfully mapped on the reference genome were extracted. As shown in Fig. S7, *M. candidum* had relatively even read depth for this segment and very few read pairs with only one read successfully mapped, while for *M. normale*, very low read depth (< 15 x) was observed from the nucleotide positions 511700 to 517800 of scaffold27 and a large number of read pairs with only one read successfully mapped were found around the nucleotide positions 507150, 511700 and 517800 of scaffold27. This suggests structural variation occurred around the three positions in *M. normale*, relative to *M. candidum*. It was thus predicted that, compared with *M. candidum*, there is a large indel or an inversion around 507150 and a large indel or high sequence divergence between 511700 and 517800 in *M. normale*.

To test this prediction, the paired-end reads with only one read successfully mapped around 507150 for the Pool-seq sample of the White morph in Dishuiyan were de novo assembled and a 655 bp-long contig was obtained. Blast search against the reference genome of *M. candidum* showed that the middle part of this contig (261-454) has no hit, but the beginning 260 bp and the ending 201 bp match well with copy1 region of the reference genome. Thus a ~200 bp insertion around 507150 was confirmed to occur in the genome of *M. normale* compared with *M. candidum*. This insertion lies in about 400 bp upstream of the start codon of *myb114* in *M. normale* (Fig. S11) and Blast search against GenBank showed that the ~200 bp sequence has no hit. PCR primers anchoring the flanking region of copy2 (F1 and R1) and one pair of internal primers (F2 and R2) were designed to infer structural variations around 511700 to 517800 in *M. normale* (Table S12; Fig. S11). The expected 1 Kb and 2.5 Kb bands were successfully amplified in one individual of *M. candidum* with the primer combination F1 and R2, and F2 and R1, respectively, but the expected 7.3 Kb band was not detected after PCR with F1 and R1. The latter might be caused by low efficiency of long fragment PCR. Five pairs of internal primers (F3 and R3, F4 and R4, F5 and R5, F6 and R6, F7 and R7; see Table S12; Fig. S11) were further designed and the internal part was then successfully amplified. Sanger sequencing of these bands indicated that they match perfectly with copy2 in sequence. In contrast, PCR amplification with F1 and R1 in an individual of *M. normale* produced a 1.3 Kb band, and no amplified bands were detected for F1 and R2, and F2 and R1. This indicated that a ~6 Kb deletion occurred in this region relative to *M. candidum*. Sanger sequencing of the 1.3 Kb band showed that the beginning part (3-135: ~130 bp) and the ending part (723-1300: ~580 bp) matched well with 511561-511696 (identity = 97%) and 518285-518796 (identity = 90%) of scaffold27, respectively, while the middle part (136-722) had no hit with this region of *M. candidum*. Therefore, most of the copy2 region in *M. candidum* has no corresponding sequence in *M. normale* due to the missing 6 Kb sequence (Fig. S11). In summary, unlike *M. candidum* which has two highly similar copies of *myb114* in the genome, *M. normale* has only one copy, *myb114-1* (we called *myb114* for short in *M. normale* hereafter since it has only one copy).

In addition, PCR amplifications were conducted with the primer combination F1 and R2, and F2 and R1, respectively, in four other species of *Melastoma* and it was found that *M. penicillatum* and *M. sanguineum* also contain two copies of *myb114*, while *M. dodecandrum* and *M. malabathricum* has the same single copy as *M. normale*. Blast searches were done for the draft genome assemblies of *M. sanguineum* and *M. dodecandrum*, which were available very recently (<http://evolution.sysu.edu.cn/Sequences.html>), using the 6 Kb highly differentiated region as the query, and it was found that two areas in one scaffold of *M. sanguineum* (2.3 and 4.2 Kb, scaffold100: 172834-175116 and 159375-163612) had 94% and 96% identities, and only one area in *M. dodecandrum* (4.2 Kb, scaffold005950: 79309-83553) had a 96% identity, with areas within the 6 Kb interval. In *M. sanguineum*, the two areas are about 11 Kb away from each other. This further confirmed that *M. dodecandrum* and *M. sanguineum* had one and two copies of *myb114*, respectively.
